# Stakeholder Perspectives and Perceived Usability of an AI‐Enabled Stroke Rehabilitation Platform: A Multicentre Mixed‐Methods Focus Group Study With Stroke Survivors, Caregivers, and Clinicians

**DOI:** 10.1111/hex.70947

**Published:** 2026-10-29

**Authors:** Poeiti Abi Saab, Stéphane Mandigout, Laura Danesin, Francesca Burgio, Marina Serena, Iñigo Chivite, Raffaele Fiorillo, Judit López, Clara Szymanski, Diana Chira, Anamaria Truță, Ștefan Strilciuc, Soléane Vielotte, Jean‐Christophe Daviet, Anna Mura, Paul Verschure

**Affiliations:** ^1^ University Limoges, HAVAE, UR 20217 Limoges France; ^2^ Neuropsychology Lab, IRCCS San Camillo Hospital Venice Italy; ^3^ Parc Sanitari Sant Joan de Déu Sant Boi de Llobregat Spain; ^4^ Institut de Recerca Sant Joan de Déu Esplugues de Llobregat Spain; ^5^ RoNeuro Institute for Neurological Research and Diagnostics Cluj‐Napoca Romania; ^6^ Department of Neuroscience Iuliu Haţieganu University of Medicine and Pharmacy Cluj‐Napoca Romania; ^7^ Research Center for Functional Genomics, Biomedicine and Translational Medicine Iuliu Haţieganu University of Medicine and Pharmacy Cluj‐Napoca Romania; ^8^ CHU Limoges, Department PMR Limoges France; ^9^ University Miguel Hernandez, UMH‐CSIC Elche Spain

**Keywords:** AI driven health care, digital health, rehabilitation, stroke, usability, user‐centred design

## Abstract

**Introduction:**

AI‐enabled digital neurorehabilitation may support home‐based stroke recovery, but its adoption depends on user acceptance. Within the European AISN project (Integrating Artificial Intelligence in Stroke Neurorehabilitation), we explored how stroke survivors, caregivers and healthcare professionals perceived the usability of a preliminary version of an AI‐based rehabilitation platform, and the barriers to its adoption.

**Methods:**

We conducted a convergent parallel mixed‐methods study in four European countries (Spain, Italy, France and Romania). Seventeen focus groups combined hands‐on testing of the platform components (RGS ecosystem and MIMS dashboard) with semi‐structured discussions. Usability was measured with the System Usability Scale (SUS), using exploratory analyses: stroke survivors and caregivers rated the RGS, whereas healthcare professionals rated the combined MIMS/RGS system. Qualitative data were analysed thematically and integrated with SUS total and item scores in a joint display.

**Results:**

Seventy‐one participants took part (27 stroke survivors, 17 caregivers, and 27 healthcare professionals). The mean SUS score was 68.1 ± 16.0 (grade C). Stroke survivors rated the patient interface (RGS) lower than caregivers rating the same interface (58.9 ± 16.5 vs. 75.6 ± 13.3; *p* = 0.002); professionals, who rated the clinician‐facing components (RGS/MIMS) after a demonstration, scored 72.7 ± 12.8. Six themes emerged: ease of use and clinical utility, personalised treatment and feedback, technical reliability and trust, the digital literacy divide, the need for human mediation and workflow and socio‐economic integration. Patients' concerns about reliability converged with their lower confidence in using the system (SUS item 9), whereas digital literacy, although described as a major barrier, was not associated with patients' scores. Human mediation, clinician time and reimbursement emerged as barriers not captured by the SUS.

**Discussion:**

Autonomous home use does not yet appear realistic for stroke survivors without support. All stakeholder groups favoured a hybrid model combining AI‐driven exercise delivery with clinician oversight. These findings are informing the refinement of the AISN platform and the design of a forthcoming randomised controlled trial.

**Patient or Public Contribution:**

Stroke survivors (*n* = 27), formal and informal caregivers (*n* = 17), and multidisciplinary healthcare professionals (*n* = 27) participated in this study as relevant stakeholders. Their contribution was central to the formative evaluation of the AISN platform, including hands‐on testing and discussion sessions focused on the RGS ecosystem and MIMS dashboard. Patients and caregivers contributed their lived experiences, expectations, concerns, and priorities regarding AI‐assisted home rehabilitation, while healthcare professionals provided their clinical expertise, including perspectives on clinical utility and workflow integration. Stakeholders were not involved in the conceptual design of the focus group (FG) study protocol, the systematic analysis of the qualitative data, or the drafting and dissemination of the manuscript. Nevertheless, their feedback served as a cornerstone of the iterative development of the AISN system. The insights and themes identified through their contributions are directly informing final technical refinements to the AISN system architecture and the protocol design for the upcoming randomised controlled trial (RCT), helping to ensure that end‐user perspectives are incorporated into the final clinical solution. While the FG script was not formally piloted with a Patient and Public Involvement and Engagement (PPIE) group, it was reviewed through multidisciplinary workshops to ensure language accessibility and clinical safety. Formal PPIE was therefore not implemented as a separate research partnership activity because stakeholders were recruited primarily as participants in a formative usability evaluation, with their experiential and professional perspectives collected as study data rather than as contributions to research design, analysis, or manuscript preparation.

## 1 Introduction

Stroke remains a primary contributor to the burden of long‐term disability worldwide, representing the second leading cause of mortality and the third leading cause of disability [1]. In Europe, approximately 1.1 million individuals suffer a stroke each year, with 9.5 million survivors living with the chronic consequences of the disease [2]. Demographic projections indicate that the number of survivors will increase by 35% by 2030 due to an ageing population, creating a severe sustainability crisis for healthcare systems [3].

While clinical guidelines emphasise the necessity of intensive, long‐term interventions to promote natural recovery (e.g., neuroplasticity), many patients experience a ‘therapeutic gap’ following hospital discharge [2, 4, 5]. Across Europe, access to high‐quality neurorehabilitation is characterised by profound regional inequalities [3], marked by significant rehabilitation workforce shortages, protracted waiting times and geographic barriers to specialised centres [2]. In response, European digital health policies, such as the Action Plan for Stroke in Europe 2018–2030, advocate for a paradigm shift toward decentralised, scalable care models that extend rehabilitation into the home [3].

Within this context, artificial intelligence (AI) and digital technologies represent a critical mechanism to bridge the service gap. AI‐enabled systems have the potential to provide personalised, objective and adaptive interventions by monitoring patient performance and optimising therapy parameters [6, 7]. However, the successful deployment of these technologies pivots not solely on technical performance (on back‐end algorithmic accuracy) but critically on front‐end user acceptance, trust, digital literacy and clinical workflow compatibility across diverse healthcare ecosystems [8, 9, 10]. Indeed, regional variability in digital literacy, cultural context and clinical practices across European countries [11] may affect how AI tools are perceived and used [12].

Despite the promise of AI‐driven rehabilitation solutions, empirical evidence regarding stakeholder acceptance of these multi‐component systems during early development remains scarce [7]. To mitigate adoption risks and ensure clinical feasibility, the development of multi‐layered AI architectures demands a structured, step‐wise co‐design approach. Engaging key stakeholders (patients, caregivers and healthcare professionals) early in the co‐design process is therefore vital to ensure that these digital tools are tailored to real‐world needs [13], fostering long‐term engagement and therapeutic adherence [8, 9, 10].

### 1.1 Study Objective

This study was conducted within the context of the European initiative AISN (Integrating Artificial Intelligence in Stroke Neurorehabilitation). The core end‐user entry points consist of the patient‐facing virtual reality‐based Rehabilitation Gaming System applications RGSapp, RGSweb, RGSclinic (delivering gamified exercises), RGSwear (for paretic arm monitoring) and the Medical Information Management System (MIMS) dashboard for clinicians' management of patients (prescription and follow‐up).

The primary objectives of this study were to: (i) assess stakeholder interaction with, and conceptual acceptance of, the RGS ecosystem patient interface and the MIMS clinical dashboard (incorporating preliminary versions of a decision‐support system for clinicians (CDSS) and a probabilistic prognostic patient model); (ii) elicit stakeholders' expectations, concerns, facilitators and obstacles to adoption, adherence and clinical integration; (iii) generate formative usability signals to refine the AISN system architecture and inform the protocol design for a forthcoming multicentre randomised controlled trial (RCT); and (iv) examine how qualitative and quantitative findings converge, complement or diverge.

## 2 Methods

### 2.1 Study Design

We conducted a multi‐centre study using a convergent parallel mixed‐methods design across four European countries (Spain, Italy, France and Romania), capturing perspectives from stroke survivors across subacute and chronic stages, their formal or informal caregivers and multidisciplinary healthcare professionals. The Focus Group (FG) format was selected to promote interaction and the co‐construction of ideas regarding user needs, preferences, and concerns [14]. To provide a comprehensive understanding of adoption barriers, the study integrated qualitative inductive thematic analysis [15] with quantitative usability benchmarking via the System Usability Scale (SUS) [16]. The rationale for this design was to examine whether, and how, the qualitative themes emerging from the FG converged with, complemented, or diverged from the quantitative usability signals (SUS) [17]. This approach also follows recent recommendations for digital rehabilitation technologies: a COSMIN systematic review found sufficient evidence for the construct validity of the SUS in this field, but no evaluation of its content validity, and recommended that SUS scores be interpreted together with qualitative data such as FGs [18]. Given the sample size, the quantitative strand was descriptive and exploratory, and the qualitative strand was considered the primary source of findings.

While the study protocol initially targeted 10 FG, planning for one FG of 5 participants per stakeholder category per country, the final implementation was expanded to 17 FG across the four European sites (Spain, Italy, Romania and France). The expansion of field implementation was driven by the feasibility of conducting additional FG sessions in France and Italy beyond the initial protocol. These additional sessions contributed to reaching data saturation across the diverse stakeholder perspectives.

### 2.2 Population, Recruitment and Eligibility Criteria

We targeted three primary stakeholder groups: (i) stroke survivors, (ii) formal or informal caregivers and (iii) healthcare professionals with experience in stroke rehabilitation. Participants were recruited using a purposive sampling strategy to ensure a diverse representation of stakeholders across four European healthcare systems.

#### 2.2.1 Recruitment Process

Clinicians at each centre identified eligible stroke survivors during routine follow‐up visits or inpatient stays. Caregivers were recruited via patient referral or through centre‐specific support network databases. Healthcare professionals were invited by site investigators (email or in‐person) based on their clinical roles and expertise. While recruitment was standardised, the Romanian site focused exclusively on stroke survivors due to local administrative and logistical constraints during the study window. Consequently, while survivor perspectives were triangulated across all four sites, caregiver and healthcare professional findings are derived exclusively from the Spanish, Italian and French centres.

#### 2.2.2 Eligibility Criteria

Participants were eligible for inclusion if they were 18 years old or older and fell into one of the three targeted stakeholder categories.

##### 2.2.2.1 Stroke Survivors

*Inclusion criteria*: (i) confirmed diagnosis of stroke (ischaemic or haemorrhagic); (ii) subacute phase (14 days to 6 months post‐stroke) or chronic phase (> 6 months post‐stroke); and (iii) functional capacity to sit in a chair or wheelchair for the duration of the session.

*Exclusion criteria:* (i) severe cognitive impairment (clinically equivalent to a Montreal Cognitive Assessment score < 19); (ii) global communication disorders (e.g., severe sensory or Wernicke's aphasia) that would preclude participation in a group discussion; (iii) severe visual impairment, or severe spasticity (clinically equivalent to a Modified Ashworth Scale score ≥ 3 in the affected upper limb), that would prevent any interaction with the digital interface. Importantly, eligibility regarding cognitive, communication and motor functions was determined through clinical judgement by site‐specific staff (physiotherapists and psychologists) based on recent medical history and routine consultations, rather than through the systematic administration of validated assessment tools.

##### 2.2.2.2 Healthcare Professionals

*Inclusion criteria:* (i) a minimum of 1 year of clinical experience working with stroke patients; and (ii) at least basic familiarity with digital health tools (e.g., electronic health records, telerehabilitation platforms, or wearable sensors).

##### 2.2.2.3 Caregivers

*Inclusion criteria:* (i) acting as a formal or informal caregiver (spouse, family member, or close contact) for a stroke survivor; and (ii) a willingness to share perspectives on the burden of care and the potential for home‐based technological support.

#### 2.2.3 Pragmatic Exclusion

Across all groups, a pragmatic exclusion was applied to those unable to use the RGS during the hands‐on session. While necessary for collecting immediate usability data, the researchers of this study acknowledge that this may introduce a selection bias towards participants with higher digital readiness.

### 2.3 Refusal Rate and Data Saturation

A methodological limitation of this study is that a formal register of individuals who declined participation or were excluded during the initial clinical screening was not systematically maintained. Consequently, the specific refusal rate is unavailable. This lack of tracking is acknowledged as a limitation, as it precludes a detailed analysis of selection bias and limits our understanding of the barriers (such as scheduling or transportation) that may have prevented certain profiles from participating in the study.

Recruitment and sampling continued iteratively within each category (five participants per FG) until saturation was reached, at which point no further participants were included.

### 2.4 Intervention Delivery Tools

Participants were presented with the physical and digital components of the AISN platform as stimuli [14].

The Rehabilitation Gaming System (Figure 1) is a virtual environment used to deliver tailored motor and cognitive gamified exercises across multiple platforms (smartphone, computer, or smartwatch) [19, 20]:

- *RGSapp*: a mobile/tablet application (Android/iOS) for gamified motor and cognitive training using virtual reality (VR) and augmented reality (AR) exercises.
- *RGSclinic*: a specialised clinical workstation featuring a high‐resolution touchscreen and advanced optical sensors (Azure Kinect DK and Leap Motion Controller) for precision upper body motion capture.
- *RGSwear and RGSweb*: a smartwatch‐based solution (compatible with Apple Watch, TicWatch Pro and Fossil) for the continuous monitoring and feedback of the paretic arm and a browser‐based platform (e.g., Google Chrome), with a standard webcam‐based tracking system for home rehabilitation. RGSwear and RGSweb were presented to participants but were not tested during the hands‐on sessions.

**Figure 1:**
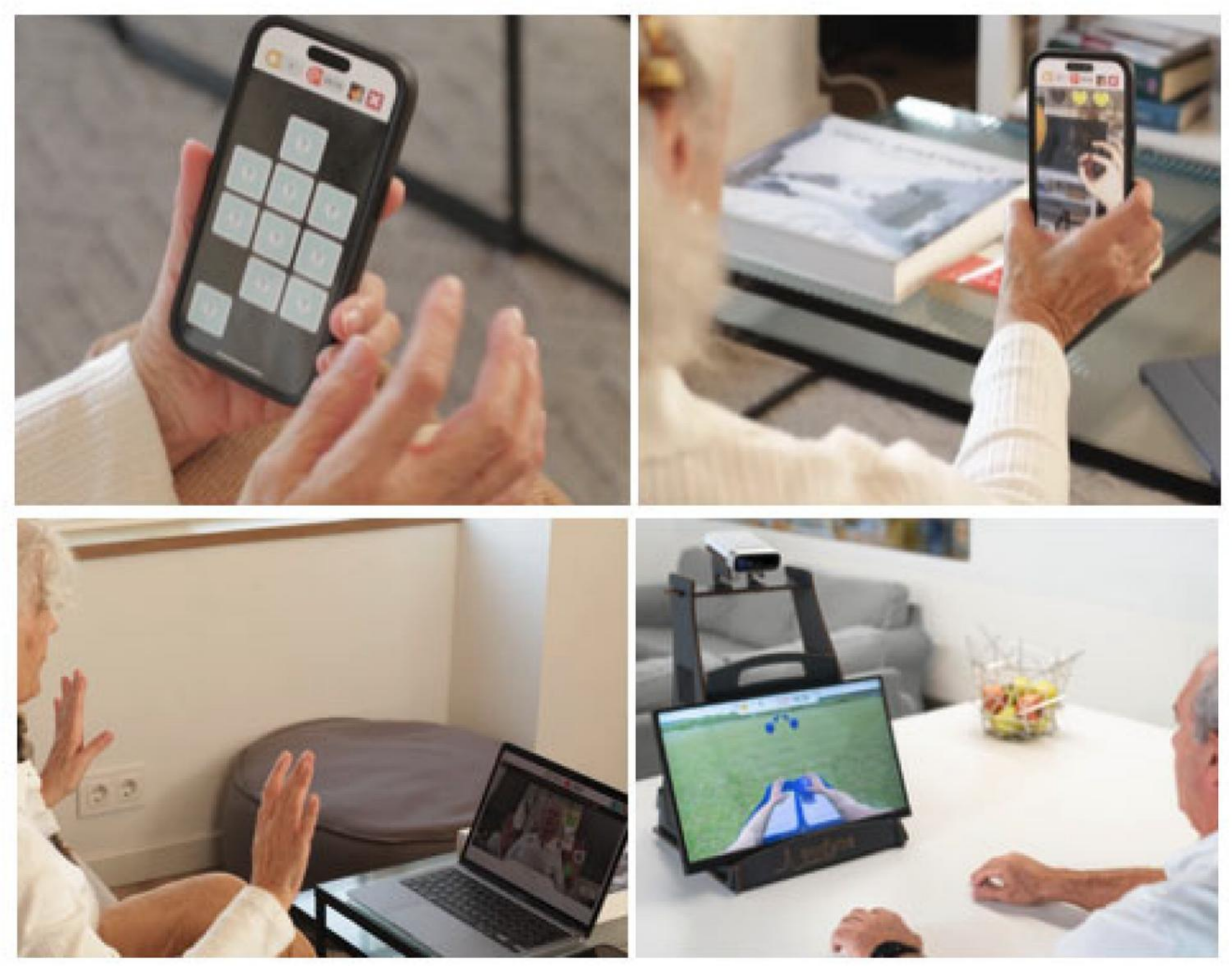
The RGSapp (top), RGSweb (bottom left), and RGSclinic (bottom right) provide cognitive and motor exercises for rehabilitation after stroke at home and the clinic.

Clinician management interface: MIMS (Figure 2) is a secure web‐based dashboard allowing clinicians to remotely manage patient profiles, create training prescriptions, track adherence and review performance reports. Interactive demonstrations and mockups were used to illustrate the integration of the deterministic clinical decision support system (CDSS) recommendation engine and probabilistic prognostic models into the MIMS workflow.

**Figure 2:**
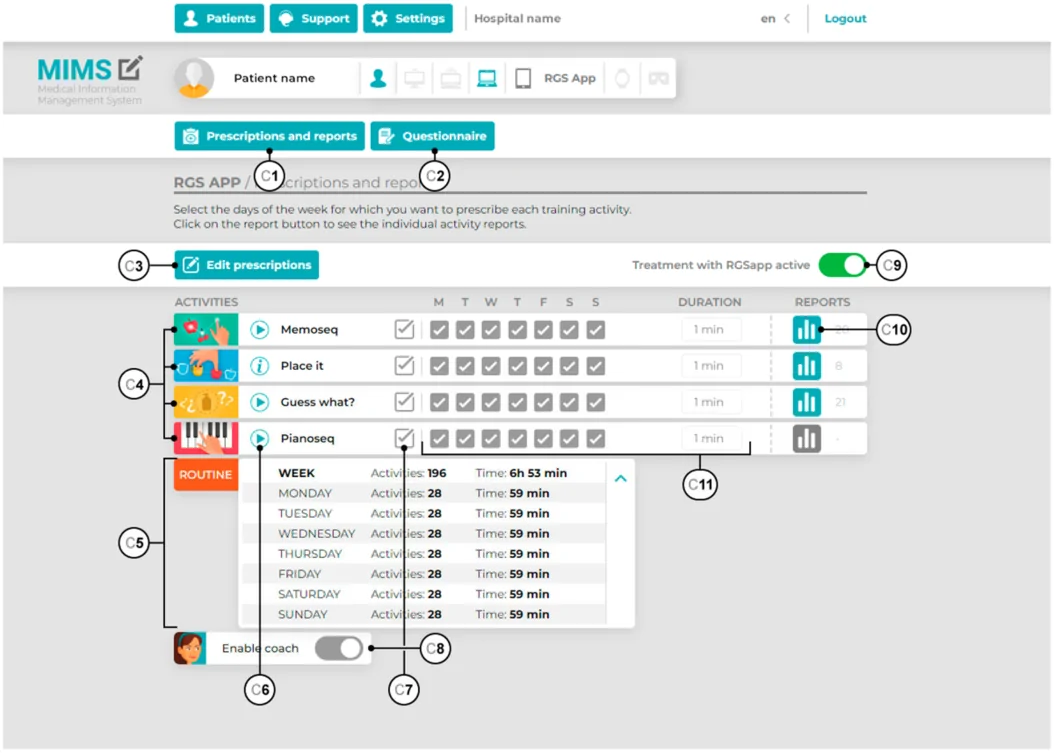
MIMS dashboard: Activity and Prescription protocol in MIMS interface: C1. Prescriptions and Reports button → Redirects to the Prescription screen. C2. Questionnaire button → Redirects to the Questionnaire screen. C3. Edit prescription → To create or edit prescriptions, click the ‘Edit Prescriptions’ button. Then click the ‘Save’ button to save the suggested changes. C4. Activity List. C5. Routine → Weekly and daily summary of prescribed activities and minutes. C6. Video/Information button → View the video (if available) and activity description. C7. Prescribe ‘Every Day’ checkbox → Prescribe a protocol for every day of the week. C8. Coach button → enable coach during the RGS session. C9. Pause/Resume Treatment toggle → Pauses or resumes an app's prescription. During the break, the patient's lack of activity will not affect adherence metrics. C10. Reports button → Access patient reports for each activity. C11. Prescription → Select the days, activities, and duration to create a prescription.

### 2.5 Scope of Evaluation

This study was conducted as a formative needs assessment and usability evaluation during the active technical development phase of the AISN platform:

1. *Active system tested*: participants interacted with (i) the RGS interfaces (executing prescribed rehabilitation exercises). RGS implements a closed‐loop architecture that uses heuristic and Bayesian algorithms to monitor performance and dynamically adjust exercise difficulty (e.g., speed, range of motion) to the user's specific needs; (ii) the baseline MIMS dashboard (managing patient profiles, adherence tracking, and manual prescription workflows).
2. *Conceptual AI requirement gathering*: participating clinicians were presented the recommendation module (CDSS) for automated exercise recommendations, and the prognostic recovery model for forecasting long‐term recovery, in their ongoing developing stage. This allowed a discussion on clinical requirements, decision‐making preferences, and trust prerequisites that directly informed the ongoing algorithmic architecture and user interface integration of these AI engines.

Gathering stakeholder requirements at this stage was intended to allow user feedback to shape the functional architecture and interface design of these AI modules prior to live clinical deployment.

This study grounded its evaluation in specific user interface entry points: the RGS ecosystem for stroke survivors and caregivers, and the MIMS dashboard interface for clinicians. Examining whether stroke survivors could interact with the RGS provided a first test of the downstream execution layer of the AISN pipeline. For healthcare professionals, the CDSS integrated into the MIMS was demonstrated, but not used, to allow clinicians to assess its potential utility. Framing the evaluation around these interface entry points allowed healthcare professionals to discuss the potential clinical utility of automated decision‐making and their requirements for transparency. To ensure that downstream AI capabilities align with real‐world clinical needs, stakeholder input was gathered *prior* to finalising the system's backend AI engines, specifically a *deterministic* CDSS for automated exercise recommendation and a *probabilistic* prognostic model for forecasting recovery trajectories. Conducting a formative evaluation of the primary interface layers at this stage allowed user feedback to contribute to shaping the functional requirements, workflow logic and design parameters of these AI modules before full technical deployment.

### 2.6 FG Procedures

#### 2.6.1 Standardisation and Harmonisation

To maintain methodological consistency across the four European clinical sites, facilitators participated in pre‐study harmonisation workshops to align moderation techniques and data assessment protocols. FG session structures were standardised into two sequential parts: Part 1: Hands‐on technical session and Part 2: Semi‐structured group interview (Figure 3). Demonstration of the technology followed a minimalist approach: facilitators provided an initial technical assistance, but transitioned to an observational role during actual interaction to capture authentic usability friction and user errors. FG scripts were translated into local languages (Spanish, Italian, Romanian and French). While not formally piloted with a Patient and Public Involvement and Engagement group, the script was validated through inter‐site harmonisation workshops involving multidisciplinary experts to ensure language accessibility and clinical safety. The AISN script is available as Supporting Information (S1).

**Figure 3:**
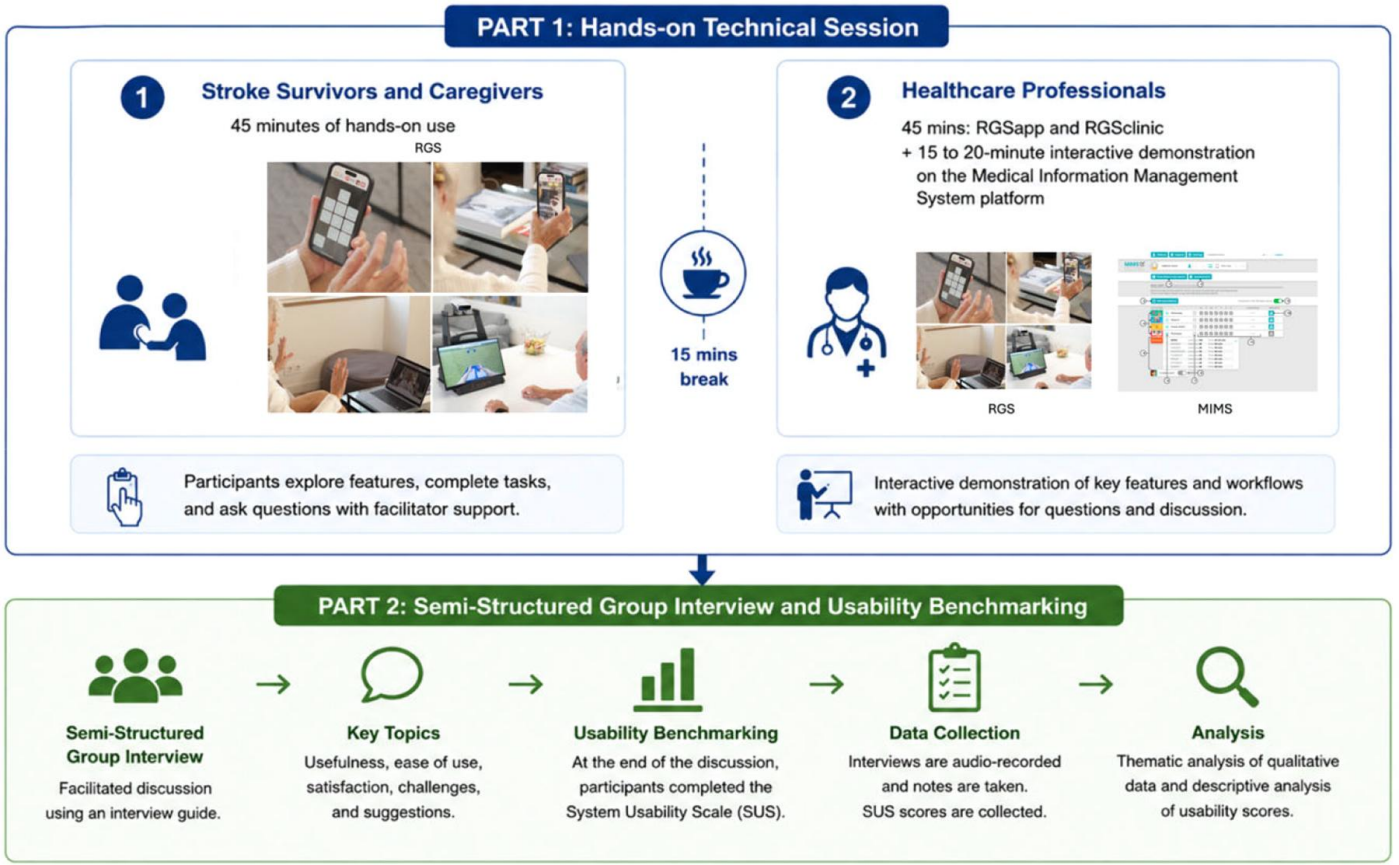
Study protocol overview.

#### 2.6.2 Part 1: Hands‐On Technical Session

Participants interacted with the AISN platform to generate authentic usability feedback.

1. *Stroke survivors and caregivers:* Engaged in 45 min of hands‐on use of the RGSapp and RGSclinic, performing gamified rehabilitation exercises with motor and cognitive tasks (such as ‘Memoseq’, ‘Blob’, ‘Shelves’, ‘Driving’, ‘Spheroids’, ‘Clean the Table’, ‘Pinball’ and ‘Hockey’). The description of exercises is available as Supporting Information (S2 and S3).
2. *Healthcare professionals:* in addition to the 45‐min hands‐on evaluation of the patient‐facing RGS, healthcare professionals were provided with a 15–20‐min interactive demonstration of the clinician‐facing MIMS dashboard. To ensure a realistic evaluation, clinicians were provided with access to the MIMS (via computer or tablet) through a secure platform managed by the AISN technical partner, Eodyne Systems S.L. The CDSS and the prognostic model were shown as demonstrations and mock‐ups; clinicians did not use them.

#### 2.6.3 Part 2: Semi‐Structured Group Interview and Usability Benchmarking (SUS)

Following the hands‐on session, the moderator led a structured discussion using the AISN script to elicit in‐depth responses regarding expectations and concerns. In all groups, the discussion focused on the use of the RGS for home‐based rehabilitation after stroke; healthcare professionals also discussed the MIMS dashboard and its planned AI modules (CDSS and prognostic model), in the perspective of prescribing and supervising home‐based rehabilitation. At the end of the discussion, participants completed the SUS. To ensure role‐relevance, patients and caregivers rated the patient‐facing interfaces (RGS), while professionals rated the integrated clinician‐facing system (MIMS/RGS). Thus, the SUS scores of stroke survivors and caregivers refer to the RGS only, whereas those of healthcare professionals refer to the RGS and the MIMS dashboard together. Consequently, SUS scores from healthcare professionals refer to a different object, rated after a shorter exposure to the clinician‐facing components, and are not directly comparable to those of patients and caregivers.

### 2.7 Safety and Fatigue Management

Recognising the clinical vulnerability of stroke survivors and the potential cognitive burden of the activity duration (2.5 h), a 15‐min break was integrated between the hands‐on session and the interview to prevent physical and cognitive exhaustion. Sessions were hosted within clinical centres, ensuring immediate access to medical support if required, and facilitated by trained clinical professionals (e.g., psychologists, physiotherapists), authorised to pause or terminate sessions if a participant appeared overwhelmed. No participants dropped out during the sessions.

### 2.8 Data Collection and Session Materials

The FG infrastructure included standardised materials to ensure consistent data acquisition across the four European sites:

- *Hardware:* tablets, smartphones (Android/iOS), and desktop workstations were provided for the hands‐on technical sessions.
- *Documentation:* participants completed standardised demographic surveys and the SUS.
- *Recording:* each site utilised high‐quality audio and video recorders to capture discussions for verbatim transcription, alongside a field notebook for moderator observations.

### 2.9 Informed Consent and Participant Autonomy

All participants provided written informed consent prior to any data collection. Recognising the clinical vulnerability of stroke survivors, the consent process was designed for maximum accessibility. Researchers provided verbal overviews of the study objectives and used simplified language to ensure participants fully understood the protocol. Participants were explicitly informed of their right to access, modify, or withdraw their consent at any time without justification or impact on their standard clinical care. Consent also included specific permission for the audio/video recording of sessions for transcription purposes, with the assurance that all identities would be de‐identified in the final report.

### 2.10 General Data Protection Regulation (GDPR) Compliance and Data Management

Data handling and storage protocols were designed to comply with the European GDPR (Regulation [EU] 2016/679) [21]. In accordance with GDPR Article 4 [7], each clinical partner acted as an independent data controller for the data collected at their respective site. Identifiable personal data (e.g., names, contact info) were stored locally on secured clinical servers and were never shared outside the European Union or with technical consortium partners. Only de‐identified study datasets, where participants were assigned a unique study ID, were combined for the central mixed‐methods analysis. Audio recordings used for thematic analysis were deleted or archived securely following verbatim transcription into anonymized text. Data will be retained for a period of 5 years following the completion of the study to allow for scientific auditing before being permanently destroyed.

### 2.11 Research Team and Reflexivity

To ensure the trustworthiness of the findings and comply with COREQ standards, the research team adopted a reflexive stance throughout the study. FG were facilitated by a multidisciplinary team of clinical researchers (including clinical psychologists and physiotherapists) holding advanced degrees (PhD, MD or MSc). All moderators were female native speakers at their respective sites and possessed extensive experience in stroke rehabilitation and qualitative moderation.

To minimise social desirability bias, facilitators established no prior relationship with participants and explicitly stated they were not the developers of the AI algorithms, maintaining independence from the technical team. Participants were informed that the researchers were part of a European consortium and that honest feedback, both positive and negative, was essential for iterative system refinement. Nevertheless, because participants were recruited by clinicians at their own centres and sessions were held in clinical settings, some degree of social desirability bias cannot be excluded.

### 2.12 Quantitative and Qualitative Data Analysis

Demographic and clinical profiles were collected from each participant via standardised surveys (digital or paper) prior to the FG sessions. For all participants, data included age, gender, educational level and digital literacy (self‐rated on a 4‐point scale: very low, basic, medium, proficient). Additionally, for stroke survivors, we recorded the time since stroke and stroke phase (subacute vs. chronic), while for healthcare professionals, we recorded their clinical role and years of experience in stroke rehabilitation. Educational level was classified by years of education to harmonise national school systems: school (primary education or up to 8 years), professional (secondary education or up to 13 years), and higher (university‐level degree). For analysis, digital literacy was grouped into three levels (very low/basic, medium, proficient), because only two participants reported very low competency.

Statistical analysis for demographic and quantitative data was conducted using RStudio, setting the threshold for statistical significance at *p* < 0.05 for all tests.

Baseline demographics variations across countries were evaluated using Welch's one‐way analysis of variance (ANOVA) with Games–Howell's post hoc test for continuous variables; Welch‐adjusted (non‐integer) denominator degrees of freedom are reported. Given the limited sample size, associations between categorical variables were examined using Fisher's exact test, rather than Pearson's *χ* ^2^ test, since expected cell frequencies were below 5 in a substantial proportion of cells in the contingency tables. The strength of the association was quantified using Cramér's *V*, with values interpreted according to the conventional thresholds proposed by Cohen [22], adjusting the thresholds for degrees of freedom if the table was not 2 × 2.

Due to small subgroups, differences in SUS scores were compared across stakeholder groups rather than between countries, using Welch's one‐way ANOVA with Games–Howell post hoc tests; mean differences are reported with 95% confidence intervals (CIs), and eta squared (*η* ^2^) as effect size. The same procedure was used to explore associations between SUS scores and participants' educational level and digital literacy, and Kendall's rank correlation (*τ*) was used for the association between age and SUS scores. Because educational level, digital literacy, and age differed markedly between stakeholder groups (e.g., all healthcare professionals had a university degree), these associations were examined across all participants and then separately within each stakeholder group; pooled results were interpreted as confounded by group membership. To support integration, mean scores on individual SUS items were also compared across groups, using Welch's *t*‐tests between stroke survivors and caregivers, who rated the same interface. Given the small subgroup sizes, all quantitative analyses were considered exploratory: no correction for multiple comparisons was applied, and *p*‐values are reported as descriptive indicators rather than as confirmatory evidence.

#### 2.12.1 Quantitative Usability

The usability of the AISN platform was benchmarked using the SUS. Following the scoring procedure reported by Brooke [16], raw SUS scores were transformed to a standard 0–100 scale and graded using the curved grading scale of Sauro and Lewis [23], which ranges from A+ (84.1–100) to F (0–51.6) and on which a score of 68 corresponds to the average (grade C). When one item was left unanswered (two participants), the total score was prorated from the nine answered items. Because the content and structural validity of the SUS have not yet been established for digital rehabilitation technologies [18], SUS scores were interpreted alongside the qualitative findings rather than in isolation.

#### 2.12.2 Qualitative Analysis Strategy

Qualitative transcripts were analysed using an inductive thematic framework. A decentralised‐to‐centralised analytical model was employed (Figure 4):

1. *Transcription and local coding:* FG recordings were transcribed verbatim in their native languages (Spanish, Italian, Romanian or French). Researchers at each clinical centre were native speakers and coded the transcripts to preserve the verbal and non‐verbal nuances of the discussions. Transcripts were managed using NVivo software (Lumivero).
2. *Translation and codebook harmonisation:* to ensure feasibility and cross‐centre alignment, researchers translated preliminary codes, thematic summaries and representative verbatim quotes into English. A standardised codebook was developed to define each code and ensure consistency in application across centres. The summary of codes is available as Supporting Information (S7–S10).
3. *Cross‐centre consensus:* joint consensus meetings were held across all sites to resolve interpretive discrepancies and establish the overarching thematic framework. During these sessions, researchers compared findings across countries, resolved interpretative discrepancies and mapped the data to identify overarching patterns related to user needs and adoption barriers.

**Figure 4:**
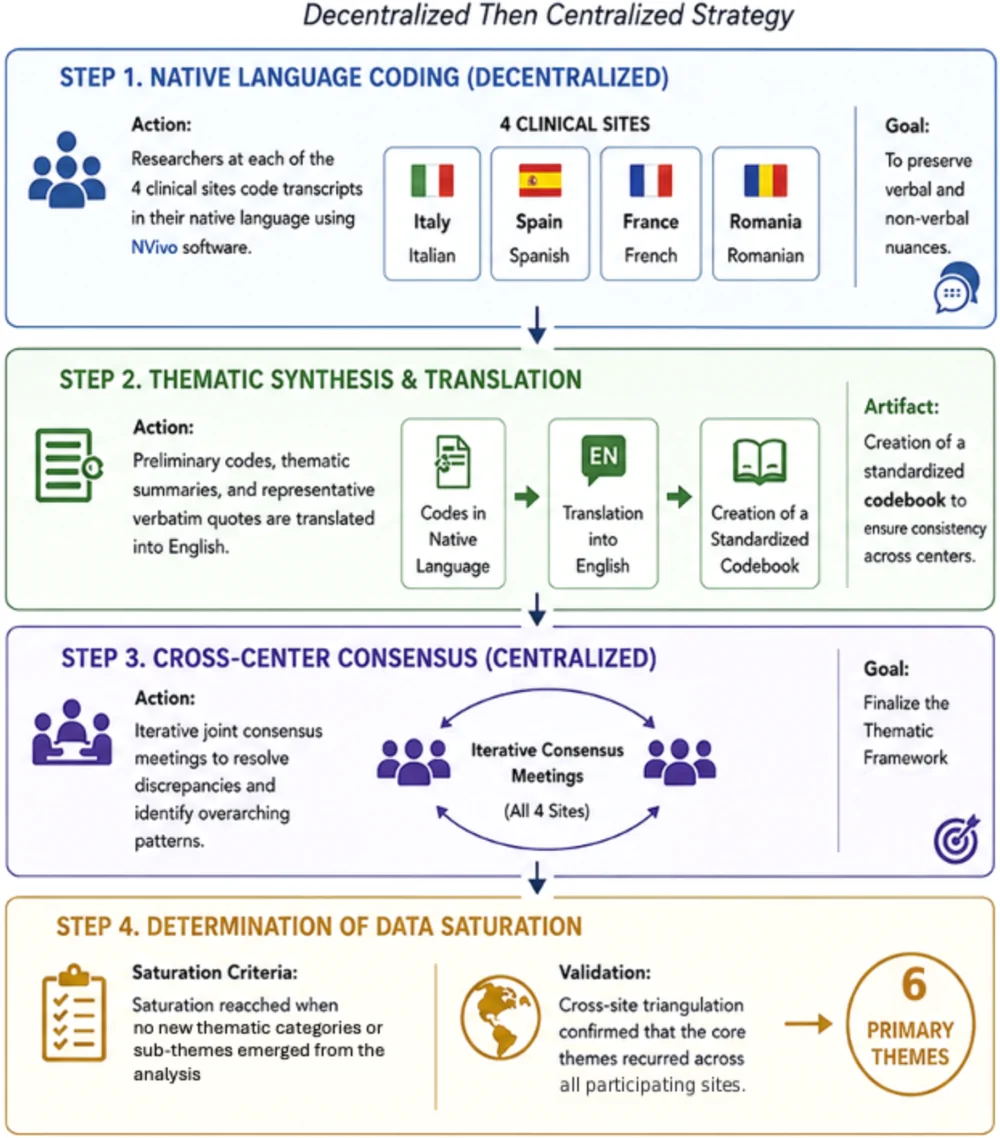
Thematic analysis flowchart (six primary themes; Romania contributed stroke survivors only).

At each centre, transcripts were coded by one researcher who was a native speaker of the language of the sessions; no independent double coding was performed. To support consistency and limit individual interpretive bias, the shared codebook, the application of codes, and the resulting themes were reviewed and discussed during the cross‐centre consensus meetings until agreement was reached, in line with a reflexive approach to thematic analysis [15].

### 2.13 Determination of Data Saturation

Data saturation was assessed at two distinct levels:

1. *Stakeholder‐group level:* data saturation was assessed separately for each participant group (patients, caregivers and healthcare professionals). FG from the different study sites were pooled within each participant group, and data collection was considered complete when no new thematic categories or sub‐themes emerged from the analysis.
2. *Cross‐geographical level:* constant comparative analysis confirmed that core themes recurred consistently across the Spanish, Italian and French sites despite cultural variations. Patients' feedback from Romania was triangulated with the patient themes identified at the other three sites, supporting the view that the main factors influencing system adoption were captured.

Although individual FG sessions were intentionally kept small to facilitate interactive discussion, a total of 17 FG were conducted across the three participant groups, providing a substantial and diverse data corpus from which the final thematic framework was developed.

Integration was performed at the interpretation stage using a joint display (Table 3). For each theme, the qualitative findings were set alongside the corresponding quantitative results, including the SUS items addressing the same construct (e.g., item 4 on the need for technical support and item 10 on the need to learn before use), and a meta‐inference was assigned: convergence (both strands point in the same direction), complementarity (each strand informs a different aspect of the phenomenon), or divergence (the strands disagree) [17].

## 3 Results

### 3.1 Participant Characteristics and Demographics

A total of 71 participants were enrolled across four European clinical sites (Italy, Spain, France and Romania). The sample comprised stroke survivors spanning subacute and chronic stages (*n* = 27), informal or formal caregivers (*n* = 17), and a multidisciplinary cohort of healthcare professionals (*n* = 27). Demographic details are provided in Table 1.

**Table 1:** Sociodemographic and clinical characteristics of participants (*n* = 71).

Characteristics	Stroke survivors (*n* = 27)	Caregivers (*n* = 17)	Healthcare professionals (*n* = 27)
Country (*n*)			
Italy	9 (FG1: *n* = 5; FG2: *n* = 4)	8 (FG1: *n* = 3; FG2: *n* = 5)	9 (FG1: *n* = 4; FG2: *n* = 5)
Spain	5 (FG1)	3 (FG1)	5 (FG1)
France	8 (FG1: *n* = 4; FG2: *n* = 4)	6 (FG1: *n* = 3; FG2: *n* = 3)	13 (FG1: *n* = 5; FG2: *n* = 5; FG3: *n* = 3)
Romania	5 (FG1)	0	0
Gender (*n*)			
Male	17	6	12
Female	10	11	15
Age (years)			
Mean (range)	67 [33–86]	62 [20–81]	32 [21–56]
Education level (*n*)			
School/Primary	9	3	0
Professional/Secondary	11	7	0
Higher/University	5	7	27
Digital competency (*n*)			
Very low/Basic	13	6	4
Medium	10	7	14
Proficient	3	4	9
Clinical profile (stroke survivors)			
Subacute phase (14 days to 6 months)	6	—	—
Chronic phase (> 6 months)	20	—	—
Time since stroke (range)	1–360 months	—	—
Clinical experience (healthcare professionals)	—	—	Mean: 6.7 ± 7.4 years

*Note:* Data derived from multisite demographic surveys. Missing data (not reported by participants): educational level for two patients, digital literacy for one patient, and stroke phase for one patient.

### 3.2 Demographics Variations Across Sites

#### 3.2.1 Stroke Survivors

A significant difference emerged between countries concerning the age of patients (Welch's *F*[3, 12.0] = 11.0, *p* < 0.001). Specifically, Romanian patients were on average younger than Spanish (*t* = 5.79, *p* = 0.002) and Italian ones (*t* = 3.54, *p* = 0.021). Moreover, French patients were younger than Spanish ones (*t* = 3.35, *p* = 0.029), while no significant differences emerged with Italian (*t* = 1.69, *p* = 0.366) or Romanian patients (*t* = 1.32, *p* = 0.572). Similarly, no significant differences were observed in digital literacy (*p* = 0.149, Cramér's *V* = 0.47), with the majority of patients reporting basic‐medium competencies in all countries. Conversely, a significant difference emerged between countries concerning patients' gender distribution (*p* = 0.046, large effect size, Cramér's *V* = 0.56), with higher rates of male participants in the Italian and Romanian samples compared to French and Spanish ones. The detailed demographic characteristics of patients are available as Supporting Information (S4).

#### 3.2.2 Caregivers

No differences were observed between countries in age (Welch's *F*[2, 4.6] = 0.984, *p* = 0.441), nor in gender distribution (*p* = 0.128, Cramér's *V* = 0.51). Conversely, significant differences were observed concerning the caregivers' digital literacy between countries (*p* = 0.007, with a large effect size, Cramér's *V* = 0.59), with Spanish and French caregivers reporting overall basic‐medium competencies, and half of Italian caregivers reporting to be proficient in the use of technologies. The detailed demographic characteristics of caregivers are available as Supporting Information (S5).

#### 3.2.3 Healthcare Professionals

No significant differences were observed between countries in age (Welch's *F*[2, 9.1] = 1.81, *p* = 0.218) and gender (*p* = 0.347, Cramér's *V* = 0.32). Conversely, a significant difference was observed concerning the digital literacy of participants from different countries (*p* = 0.003, with a large effect size, Cramér's *V* = 0.52), with Italian healthcare professionals reporting overall lower digital competencies compared to Spanish and French ones. The detailed demographic characteristics of healthcare professionals are available as Supporting Information (S6).

### 3.3 Quantitative Usability Assessment (SUS Scores)

The AISN platform received an overall mean SUS score of 68.1 ± 16.0, corresponding to a grade C, close to the benchmark average of 68 [23]. Patients and caregivers rated the same RGS (patient‐facing) interface after 45 min of hands‐on use, while professionals rated the integrated clinician‐facing system (MIMS/RGS) after a 15–20‐min demonstration. All 71 participants completed the SUS.

SUS scores differed between stakeholder groups (Welch's *F*[2, 41.0] = 8.07, *p* = 0.001, *η* ^2^ = 0.21), with stroke survivors reporting the lowest perceived usability (Table 2). Games–Howell comparisons showed that stroke survivors scored lower than caregivers (mean difference −16.7 points, 95% CI −27.7 to −5.7, *p* = 0.002) and healthcare professionals (−13.8 points, 95% CI −23.5 to −4.1, *p* = 0.003), whereas caregivers and healthcare professionals did not differ (2.9 points, 95% CI −7.1 to 12.8, *p* = 0.76). Only the comparison between stroke survivors and caregivers concerns the same interface; the comparison with healthcare professionals reflects differences in the object rated and in exposure, as well as in stakeholder role.

**Table 2:** SUS scores breakdown by stakeholder group and site (stroke survivors and caregivers: RGS; healthcare professionals: MIMS/RGS).

Stakeholder group	Italy	Spain	France	Romania	Group mean (±SD)	Grade
Stroke survivors (*n* = 27)	56.1 ± 19.6	63.8 ± 15.6	63.4 ± 15.8	51.5 ± 12.9	58.9 ± 16.5	D
Caregivers (*n* = 17)	74.7 ± 16.3	75.0 ± 15.6	77.0 ± 9.4	N/A	75.6 ± 13.3	B
Healthcare professionals (*n* = 27)	64.2 ± 12.1	82.5 ± 5.9	74.8 ± 12.0	N/A	72.7 ± 12.8	B−
Overall (*n* = 71)					68.1 ± 16.0	C

*Note:* Grades follow the Sauro–Lewis curved grading scale [23]; a score of 68 corresponds to the average. Patients and caregivers rated the RGS; healthcare professionals rated the MIMS/RGS system. Site‐level means are descriptive: with 3–13 participants per cell, they do not support between‐site comparisons. N/A indicates no recruitment for this category at the specific site.

#### 3.3.1 Factors Associated With Usability Scores

Across all participants, SUS scores were associated with self‐rated digital literacy (Welch's *F*[2, 38.0] = 5.42, *p* = 0.008) and educational level (Welch's *F*[2, 25.4] = 5.28, *p* = 0.012): participants reporting proficient digital skills scored higher than those with basic (*t* = 2.85, *p* = 0.019) or medium digital skills (*t* = 2.89, *p* = 0.018), and participants with higher education scored higher than those with a lower educational level (*t* = 3.27, *p* = 0.012). Age was negatively correlated with SUS scores (Kendall's *τ* = −0.20, *p* = 0.018). These pooled associations are confounded by stakeholder group, since healthcare professionals were younger, all university‐educated, and reported higher digital literacy. Within stakeholder groups, none of these factors was associated with SUS scores among stroke survivors (digital literacy: Welch's *F*[2, 6.0] = 0.62, *p* = 0.567; education: Welch's *F*[2, 10.7] = 0.27, *p* = 0.772; age: Kendall's *τ* = 0.01, *p* = 0.950), and stroke survivors with scores below 51.7 (grade F, *n* = 13) did not differ from the others (*n* = 14) in age (66.8 vs. 67.3 years, *p* = 0.92), digital literacy (Fisher's exact *p* = 0.39) or education (*p* = 0.45). Among caregivers, older age was associated with lower scores (*τ* = −0.49, *p* = 0.008), whereas digital literacy (Welch's *F*[2, 7.6] = 3.36, *p* = 0.090) and education (Welch's *F*[2, 5.5] = 5.22, *p* = 0.054) did not reach significance. Among healthcare professionals, scores differed by digital literacy (Welch's *F*[2, 8.2] = 6.37, *p* = 0.021), professionals with medium competency scoring lower than proficient ones (mean difference 15.0 points, 95% CI 4.7–25.4, *p* = 0.005), but not by age (*τ* = 0.13, *p* = 0.345).

Item‐level scores (1–5 agreement scale) are reported in Supporting Information S11, and individual responses in Supporting Information S12. Compared with caregivers rating the same interface, stroke survivors were less inclined to use the system frequently (item 1: 3.41 vs. 4.47, *p* = 0.002), found it more complex (item 2: 2.93 vs. 1.65, *p* < 0.001) and more cumbersome (item 8: 2.85 vs. 1.76, *p* = 0.002), perceived more inconsistency (item 6: 2.63 vs. 1.76, *p* = 0.046), and felt less confident using it (item 9: 3.56 vs. 4.24, *p* = 0.044). They also reported a greater need for technical assistance (item 4: 3.37 vs. 2.59; healthcare professionals: 2.30), although this difference was not statistically significant (*p* = 0.10). Ratings of ease of use (item 3: 3.63 vs. 4.06), learnability (item 7: 3.59 vs. 3.76), functional integration (item 5: 3.96 vs. 4.29) and the need to learn before use (item 10: 2.78 vs. 2.82) did not differ.

### 3.4 Thematic Analysis of Qualitative FGs

The analysis of 17 FGs yielded six primary themes capturing stakeholder expectations, technical barriers and workflow requirements. The following sections present the six core themes incorporated into the analysis with quotations appropriately identified by stakeholder group and country. Quotations refer to the use of the RGS for home‐based rehabilitation after stroke, except those of healthcare professionals on remote monitoring and AI‐based recommendations (Themes 3 and 6), which refer to the MIMS dashboard and its planned AI modules (CDSS and prognostic model), considered for prescribing and supervising home‐based rehabilitation.

#### 3.4.1 Theme 1: Perceived Ease of Use and Clinical Utility

Healthcare professionals and caregivers mostly described the system as intuitive and as a valuable extension of standard care, while stroke survivors' accounts focused more on the value of the exercises for staying active. Stakeholders also emphasised that digital tools are very good complementary home exercise drivers in addition to hands‐on clinical therapy.

**Verbatim:** “The application was intuitive, very good, once familiarised with it.” (**Healthcare Professional, France**). “It's easy for patients to get involved because it's very intuitive” (**Healthcare Professional, Spain**). “I would continue to use it even after I got better physically, to keep my mind active” (**Patient, Italy**). “That [the RGS tool] can be good, because it's me who stimulates him when he doesn't have physiotherapy or speech therapy…”

(**Caregiver, France**)

#### 3.4.2 Theme 2: Importance of Personalised Treatment and Feedback

Stakeholders expressed a strong demand for tools tailored to specific motor or cognitive deficits. Clinicians and caregivers emphasised that real‐time feedback and adaptive algorithmic logic are essential to ensure training exercises are performed correctly, to maintain long‐term motivation, and to avoid cognitive overburden.

**Verbatim:** “I think we prefer the one that is adapted to our needs.” (**Patient, France**). “It is a good thing that AI learns from you in order to adapt to it.” (**Caregiver, Spain**). “Each person has lost something that is not exactly the same as the person next door, some patients may be able to use these tools, while others may have difficulty” (**Caregiver, Italy**). “I find it potentially quite interesting that the patient can adapt, and that you can adapt the treatments yourself.” (**Healthcare Professional, Spain**). “Being on your mobile all the time: no activity of daily living, no social links, no leisure activities” (**Healthcare Professional, Italy**). “Translate the game into activities of daily living”

(**Healthcare Professional, France**)

#### 3.4.3 Theme 3: Technical Reliability and Trust

Participants linked their trust in the system to its technical stability. Stroke survivors mainly raised anticipated problems, such as what to do if the application crashed or the internet connection failed, and uncertainty about coping alone at home. Caregivers raised data security and the need for visual, testable explanations, and clinicians raised the risk of over‐reliance on automated outputs.

**Verbatim:** “When it crashes, what do we do?” (**Patient, France**). “What about the lack of internet connection?” (**Patient, France**). “There is also a distrust of digital technology, particularly among older generations.” (**Patient, Italy**). “Technical support and more explanation of each example, not only written, but also visual and testable.” (**Caregiver, Spain**). “…like stealing data? Well, that can be a risk with any personal device anyway.” (**Caregiver, France**). “What about lack of internet connection?” (**Healthcare Professional, France**). “I'll be afraid of relying too much, telling myself the computer said this and going on from there or patient profile will be too standard”.

(**Healthcare Professional, France**)

#### 3.4.4 Theme 4: Digital Literacy Divide and Adoption Preparation

Participants described digital skills as a precondition for using the system. Patients and clinicians described a “digital health divide”: older patients, or those with little experience of smartphones and tablets, were expected to need ongoing assistance to use the system at home.

**Verbatim:** “Some of my patients are over 50 years old and don't know how to use mobile phones.” (**Healthcare Professional, Spain**). “What skills do patients need to use them? Many would need someone to accompany them to use the technology. They would need to be used to using the phone, for example, on a day‐to‐day basis” (**Healthcare Professional, Spain**). “I don't know how to use a tablet like that” (**Patient, Italy**). “I never do that on my phone” (**Patient, France**). “One would need at least basic digital competencies to perform the exercises” (**Patient, Romania**). “My mother didn't listen to the explanation, and then she didn't understand it”.

(**Caregiver, Italy**)

#### 3.4.5 Theme 5: The Need for Human Interaction (Hybrid Care Model)

This theme reflects a requirement for hybrid care rather than a resistance to technology. Participants in all three stakeholder groups rejected a fully automated model, viewing the therapist's emotional support and clinical reassurance as irreplaceable motivators.

**Verbatim:** “Human contact and professional‐patient interaction at the level of motivation, both positive and negative, is irreplaceable. Especially at the beginning, and if a failure is detected. Human interaction even to detect if something is not detecting well” (**Healthcare Professional, Spain**). “Patients need human help” (**Healthcare Professional, France**). “Constant contact with the therapist is fundamental for the rehabilitation success” (**Patient, Romania**). “Contact is essential” (**Caregiver, France**). “When I hear about it, I think it's to eliminate contact”

(**Caregiver, Italy**)

#### 3.4.6 Theme 6: Clinical Workflow and Socio‐Economic Integration

Successful adoption depends on how well the system integrates into the existing clinical and social infrastructure. Key concerns included the time burden for clinicians to monitor data and the financial accessibility (reimbursement) for patients following discharge.

**Verbatim:** “The biggest constraint we have is time. To see the patient, to collect the data and to nurture the platform.” (**Healthcare Professional, Spain**). “The possibility to monitor patients' performances remotely is a strength.” (**Healthcare Professional, Italy**). “All these are tools, and this could be integrated as a complementary tool.” (**Patient, France**). “For me, the crux of the matter is how we finance it.” (**Caregiver, France**). “It's always a question of budget.”.

(**Caregiver, Italy**)

### 3.5 Mixed‐Methods Integration: Convergence, Complementarity and Divergence

Table 3 sets each qualitative theme alongside the corresponding quantitative findings and states the resulting meta‐inference.

**Table 3:** Joint display of qualitative themes and quantitative usability findings.

Theme	Qualitative finding	Quantitative finding	Meta‐inference
1. Perceived ease of use and clinical utility	Clinicians and caregivers described the RGS as intuitive; patients' accounts focused on the value of the exercises.	Patients 58.9 versus caregivers 75.6, same interface (*p* = 0.002). Complexity (item 2) 2.93 versus 1.65; cumbersome (item 8) 2.85 versus 1.76; ease of use (item 3) 3.63 versus 4.06; learnability (item 7) 3.59 versus 3.76.	**Divergence**: the ease of use described by caregivers and clinicians was not reflected in patients' ratings; the gap lay mainly in perceived complexity and cumbersomeness.
2. Personalised treatment and feedback	All groups valued adaptive difficulty; clinicians warned against screen time replacing daily‐life activities.	Not captured by the SUS.	**Complementarity**: the qualitative data identify a requirement that usability scores do not measure.
3. Technical reliability and trust	Patients anticipated crashes and connectivity problems and were uncertain about coping alone.	Confidence (item 9) 3.56 versus 4.24 for caregivers (*p* = 0.044); inconsistency (item 6) 2.63 versus 1.76 (*p* = 0.046); integration (item 5) 3.96 versus 4.29.	**Convergence**: patients' lower confidence and greater perceived inconsistency match their anticipated concerns about reliability and coping alone.
4. Digital literacy divide	Patients and clinicians described limited phone/tablet skills as a barrier to autonomous use.	Pooled: digital literacy *p* = 0.008, age τ = −0.20 (confounded by group). Within patients: no association with digital literacy (*p* = 0.57), education (*p* = 0.77) or age (τ = 0.01). Need to learn (item 10): patients 2.78, caregivers 2.82, professionals 1.85.	**Divergence**: digital skills were a major concern in the discussions, but within the patient group they were not associated with SUS scores; the pooled associations reflect group composition.
5. Need for human mediation	All groups considered the therapist irreplaceable, especially at the start of home use.	Need for assistance (item 4): patients 3.37, caregivers 2.59 (*p* = 0.10), professionals 2.30. Caregivers' SUS 75.6 (grade B).	**Convergence** for patients, whose need for assistance was highest; **complementarity** for caregivers, whose good usability ratings did not remove the demand for clinician involvement.
6. Clinical workflow and socio‐economic integration	Clinicians valued remote monitoring but named time as the main constraint; caregivers raised cost and reimbursement.	Clinicians' SUS 72.7 (grade B−), rating the MIMS/RGS after a demonstration.	**Complementarity**: the SUS does not capture time burden or funding, the main barriers raised.

*Note:* SUS results of stroke survivors and caregivers refer to the RGS; SUS results of healthcare professionals refer to the MIMS/RGS system. Qualitative findings refer to the use of the RGS for home‐based rehabilitation, except clinicians' views on remote monitoring and AI‐based recommendations (MIMS).

Integration showed four patterns. First, a divergence: the ease of use described by caregivers and clinicians was not reflected in stroke survivors' ratings of the same RGS interface. Second, a convergence around confidence and support: stroke survivors felt less confident using the system (item 9) and perceived more inconsistency (item 6) than caregivers, and reported the greatest need for technical assistance (item 4), consistent with their accounts in Themes 3 and 5. Third, a divergence regarding digital skills: although participants described digital literacy as a major barrier (Theme 4), neither digital literacy, education nor age was associated with SUS scores within the patient group. Fourth, complementarity: several of the main barriers to adoption (the need for human mediation, clinician time, and reimbursement) fall outside what the SUS measures and emerged only from the qualitative data. These patterns are descriptive; the design does not allow causal links between themes and scores.

#### 3.5.1 Stakeholder Agreements and Divergences

While the thematic synthesis identified shared goals, the evaluation revealed distinct, role‐specific priorities. A consensus emerged regarding the irreplaceable role of human mediation (Theme 5) and the necessity of personalised, adaptive treatment (Theme 2). Divergences, however, were prominent in the primary barriers to adoption: stroke survivors focused on interface stability and access to their therapist when facing technical problems (Theme 3); caregivers emphasised affordability and reimbursement (Theme 6); and healthcare professionals emphasised clinical utility, workflow efficiency, and the capacity of the MIMS dashboard to support remote patient monitoring (Theme 6).

## 4 Discussion

### 4.1 Principal Findings

This multicenter study provides a stakeholder‐centred usability evaluation of the AISN platform (Integrating AI in Stroke Neurorehabilitation), engaging 71 participants across 17 FG in four European healthcare systems (Italy, Spain, France and Romania). Our findings show a tension between the perceived potential of AI‐enabled neurorehabilitation and the practical barriers to its adoption in home environments. While our qualitative findings suggest that stakeholders recognise the potential of AI‐based tools, the lower quantitative usability scores, particularly among stroke survivors, indicate that the current system is a solution still in its development stage. These results serve as formative signals for iterative refinement, a process that will be evaluated in a forthcoming multicentre RCT.

All participants completed the sessions, which lasted up to 2.5 h; this supports the feasibility of the protocol for future evaluations with stroke survivors. However, the qualitative findings emphasise that technical reliability and digital literacy were central to how participants anticipated trusting and using the system at home. Furthermore, our findings suggest that stakeholders do not view the system through a single lens; their priorities differed by role, ranging from cognitive load (patients) to administrative efficiency (clinicians). These role‐dependent perspectives indicate that future system refinements must move beyond a universal design to a multi‐layered approach that simultaneously ensures user interface (UI) reliability for patients, provides structural support for caregivers, and optimises data‐management capabilities for clinicians.

### 4.2 Divergent Usability Ratings: Triangulating Perceptions and Scores

Stroke survivors rated the RGS interface lower (58.9) than caregivers rating the same interface (75.6). The higher score of healthcare professionals (72.7) is not directly comparable, because they rated a different object (the MIMS/RGS system) after a shorter demonstration. Integration of the quantitative and qualitative data offers possible, but not causal, explanations for these differences.

Healthcare professionals primarily evaluated the system's clinical decision support features (MIMS dashboard), which they perceived as high‐value tools for remote monitoring and data management. Their scores may therefore reflect the ‘perceived utility’ of efficient clinical workflows and first impressions, rather than the physical ease of using the interface.

In contrast, stroke survivors directly interacted with the gamified RGS interface. In the discussions (Theme 3), they anticipated technical problems such as crashes or connection losses and expressed uncertainty about how they would cope alone (‘When it crashes, what do we do?’) [24]. These concerns are consistent with, but cannot be shown to account for, their lower SUS scores. At item level, patients also reported lower confidence (item 9) and a greater need for technical assistance (item 4) than caregivers rating the same interface.

Across all participants, lower self‐rated digital literacy and older age were associated with lower SUS scores, although these associations were confounded by stakeholder group; within the patient group, neither digital literacy, education nor age was associated with SUS scores, whereas older caregivers rated the system lower. In the discussions (Theme 4), patients and clinicians described a ‘digital health divide’, in which older survivors or those with limited experience of digital devices expected to need assistance [11]. Patients' concerns about digital skills may therefore relate more to anticipated autonomous use at home than to the ratings they gave after a supervised session; the role of digital familiarity in perceived usability [5, 12] should be tested in larger samples.

By combining SUS scores with the discussions, this study goes beyond stand‐alone reporting of SUS scores: it identifies possible reasons for the lower ratings among direct users, together with barriers that the SUS does not capture [21], as recommended for the evaluation of digital rehabilitation technologies [18].

Site‐level SUS scores varied descriptively (e.g., 51.5 among the five Romanian patients), but subgroups of 3–15 participants do not allow inferences about national or institutional factors. Regional differences in digital health maturity, described in the literature [3, 5], warrant investigation in larger multicentre samples.

### 4.3 From Automation to Hybrid Care: The Persistent Need for Human Mediation

A finding shared by all three stakeholder groups was the rejection of a fully automated, clinician‐free rehabilitation model. Qualitative analysis of Theme 5 (need for human interaction) suggests that improving technical usability alone would not remove the demand for a ‘human in the loop’ [25]. This finding supports a hybrid care model as a framework for the integration of AI‐based solutions [26].

Healthcare professionals and stroke survivors emphasised that the therapeutic alliance, the bond of trust and mutual goals, is important for functional recovery. Clinicians considered that, while the adaptive engine of the RGS can adjust task difficulty, it cannot replace the motivational and reassuring role of the therapist (Theme 2, Theme 5). As one professional noted, human interaction is ‘irreplaceable,’ particularly during the early stages of home‐based sessions (Theme 5).

Stakeholders' accounts are consistent with the view of AI components not as substitutes for clinical expertise, but as ‘force multipliers’ [6] that may help bridge the post‐discharge therapeutic gap. The MIMS dashboard, with the future development of an AI‐based CDSS and a prognostic model, was valued by professionals specifically because it could allow them to remain present in the patient's home via prescription management and data monitoring, maintaining the professional contribution without the need for physical travel [13]. Caregivers expressed a strong desire for home‐based digital tools to help them stimulate patients, especially when access to hospital sessions is limited. Some caregivers also asked for technical support and clearer explanations (Theme 3), and the burden of technical troubleshooting placed on families has been reported previously [27]. For a hybrid model to succeed, technical support should be handled by the clinical system, leaving the caregiver to focus on the social and motivational aspects of the recovery routine [5, 12, 28].

The variation in usability ratings with digital literacy, together with participants' demand for tailored exercises (Theme 2), is consistent with broader literature suggesting that successful adherence to self‐practice requires tailoring to one's reality rather than a standardised ‘one‐size‐fits‐all’ approach [29, 30]. AI‐driven personalisation may, therefore, need to be complemented by clinician‐led adjustments to ensure the system respects the user's specific life circumstances [4].

The transition from hospital to home (the ‘soft‐landing’) may depend on a system that leverages explicit intelligence for technical feedback while maintaining human mediation for motivation and safety [4, 31]. Participants' willingness to use such tools was consistently conditioned on their being delivered as part of a connected, clinician‐guided solution (Theme 5).

### 4.4 Study Limitations

Several methodological limitations should be considered when interpreting these results:

1. *Confounding of stakeholder group, site, and component evaluated*: patients and caregivers rated the RGS after 45 min of hands‐on use, whereas healthcare professionals rated the combined MIMS/RGS system after a 15–20‐min demonstration, and Romania contributed only patients. Differences in SUS scores between groups therefore cannot be attributed to stakeholder role alone, and scores for the clinician‐facing components reflect first impressions rather than use.
2. *Clinical scope and pragmatic exclusions*: participants were selected to match the RGS app's validated clinical indication (judged by clinicians to be clinically equivalent to MoCA ≥ 19), aligning usability benchmarks directly with its target population. However, excluding individuals unable to physically interact with the interface during hands‐on testing may introduce selection bias toward patients with higher motor function or digital readiness.
3. *Sample size and geographical generalisability*: while the total sample of 71 participants is substantial for a qualitative exploration, the small subgroups at each site (e.g., five patients in Romania or Spain) lack the statistical power for robust cross‐country comparisons. Consequently, these site‐specific observations should be interpreted with caution as formative signals rather than generalisable conclusions.
4. *Exploratory statistics*: quantitative analyses involved multiple tests on small subgroups without correction for multiple comparisons and should be regarded as hypothesis‐generating. Moreover, the content and structural validity of the SUS have not been established for digital rehabilitation technologies, and its applicability to people with cognitive or communication impairments remains unverified [18], which may have affected stroke survivors' ratings.
5. *Evaluation phase scope*: as an exploratory study, stakeholders evaluated the AISN ecosystem through hands‐on sessions and interactive demonstrations rather than through longitudinal home use. Thus, these results capture early usability signals and expectations rather than demonstrated long‐term clinical effectiveness or real‐world adherence. Patients' comments on technical failures were largely anticipatory, and technical incidents during the sessions were not systematically recorded.
6. Procedural density: the session protocol was dense, lasting up to 2.5 h and may have introduced cognitive load. Although the 100% retention rate and clinical fatigue management (mandatory breaks) support the protocol's feasibility for research, cognitive fatigue may have influenced the technical ratings from older survivors.
7. *Social desirability*: participants were recruited by clinicians at their own centres, and sessions were held in clinical settings and moderated by clinical staff, which may have encouraged favourable feedback.
8. An imbalance in stakeholder representation across countries must be acknowledged as a limitation. Specifically, Romania contributed only stroke survivors, meaning that caregiver and clinician perspectives do not incorporate Romanian cultural or institutional contexts.
9. *Single‐researcher coding*: transcripts were coded by one researcher per centre without independent double coding, which may increase the risk of interpretive bias; this was mitigated, but not eliminated, by the shared codebook and the cross‐centre consensus meetings.

### 4.5 Future Perspectives and RCT Preparation

The empirical findings from this study are used to inform the final technical refinements and the protocol design of the clinical validation study of the AI‐based ecosystem of the AISN platform. Qualitative barriers identified in Theme 3 (Technical reliability) and Theme 4 (Digital literacy) are being addressed through UI and UX (User Experience) refinements, including more intuitive features and simplified training modules for elderly users. Stakeholder requirements regarding transparency and clinical control are guiding the final integration of the *deterministic* CDSS recommendation engine and the *probabilistic* prognostic recovery informational model into the MIMS dashboard interface, in line with the transparency and human‐oversight requirements that the EU Artificial Intelligence Act sets for high‐risk AI systems [32].

The hybrid care model suggested by these findings will be tested in a forthcoming multi‐centre RCT. The RCT protocol is being designed around a hybrid care framework pairing automated exercise delivery via the RGSapp with scheduled, remote clinician monitoring via the MIMS dashboard. This model leverages the AI recommendation engine and prognostic visualisation tools to evaluate clinical efficacy, adoption dynamics, and long‐term therapeutic adherence [6]. This approach aims to bridge the ‘therapeutic gap’ while preserving the therapeutic alliance that stakeholders identified as irreplaceable (Theme 5) [23].

## 5 Conclusion

This multicentre mixed‐methods study captured early usability signals and expectations regarding the AISN platform from stroke survivors, caregivers, and clinicians. Stroke survivors rated the patient interface lower (SUS 58.9) than caregivers rating the same interface (75.6), and anticipated difficulties related to technical reliability and digital skills. Healthcare professionals, who rated the clinician‐facing components after a demonstration, valued their potential for remote monitoring (SUS 72.7).

Stakeholders in all groups rejected a fully automated rehabilitation model and advocated a hybrid care model in which AI‐enabled tools, such as the MIMS dashboard and its planned AI‐based modules, support rather than replace the therapeutic relationship.

As an exploratory study, these results reflect stakeholder perceptions and early usability signals rather than clinical outcomes. Nevertheless, the insights gathered are informing iterative technical refinements to the AISN platform. The hybrid care model and the usability of the refined system will be evaluated in a forthcoming RCT. Ultimately, the success of AI in neurorehabilitation is likely to depend on user‐centred design and iterative evaluation that balance technological and algorithmic precision with the human reassurance of the clinician‐patient relationship.

## Author Contributions

**Poeiti Abi Saab:** writing – original draft, methodology, writing – review and editing, data curation, formal analysis. **Stéphane Mandigout:** validation, supervision. **Laura Danesin:** writing – review and editing, methodology, formal analysis, data curation. **Francesca Burgio:** methodology, writing – review and editing. **Marina Serena:** writing – review and editing, methodology. **Iñigo Chivite:** methodology, writing – review and editing. **Raffaele Fiorillo:** methodology, supervision. **Judit López:** methodology, writing – review and editing. **Clara Szymanski:** methodology, writing – review and editing. **Diana Chira:** methodology, writing – review and editing, formal analysis, data curation. **Anamaria Truță:** writing – review and editing, formal analysis, data curation, methodology. **Ștefan Strilciuc:** supervision, visualisation. **Soléane Vielotte:** visualisation. **Jean‐Christophe Daviet:** visualisation. **Anna Mura:** conceptualisation, investigation, writing – review and editing, validation, methodology, formal analysis, data curation, supervision. **Paul Verschure:** conceptualisation, investigation, validation, visualisation, writing – review and editing, project administration, supervision.

## Ethics Statement

The study was conducted in strict accordance with the ethical principles of the Declaration of Helsinki. Ethical approval was obtained from the relevant Institutional Review Boards (IRB) or Ethics Committees at each of the four clinical sites prior to the commencement of recruitment. These approvals were aligned with the following national regulatory frameworks:

- Spain: approved by the Comité de Ética de la investigación con medicamentos (CEIM) at Parc Sanitari Sant Joan de Déu.
- Italy: approved by the Comitato Etico Territoriale Area Centro‐Est Veneto at the IRCCS San Camillo Hospital.
- France and Romania: approved by the local institutional Ethics Committee in accordance with national health research regulations.

## Conflicts of Interest

The authors declare no conflicts of interest.

## Supporting information


Supporting File

## Data Availability

The data underlying this study are not publicly available due to institutional, ethical, and confidentiality restrictions. Data may be available from the participating centres upon reasonable request and subject to the approval of the relevant institutions.
