# Supplementary material for: Stakeholder Perspectives and Perceived Usability of an AI‐Enabled Stroke Rehabilitation Platform: A Multicentre Mixed‐Methods Focus Group Study With Stroke Survivors, Caregivers, and Clinicians
Source: Health Expect. 2026 Oct 29;29(6):e70947. doi: 10.1111/hex.70947 (PMC13720347; doi:10.1111/hex.70947)
Supplement: Supplementary file 1 [file HEX-29-e70947-s001.docx]

**Supplementary Material**

**S1. Focus Group Script**

**AISN Treatment delivery tools: RGS system (~ 30 min)**

- How was your experience with the RGS system (RGSapp, RGSwear, RGSclinic)? Did you find it easy to use
- How did you feel while using this type of technology?
- Would you like to use this technology for rehabilitation at home? Why / why not?
- Would you feel confident performing rehabilitation at home with this technology?
- What kind of support do you think you might need to do home rehabilitation with this technology
- Do you think that performing rehabilitation at home could imply some risks for the patients? Which ones?
- Do you think you have enough technical/technological knowledge to use RGS on your own
- Would you like to use this technology regularly? Why? Or why not
- What do you think about using the RGSclinic, RGSapp and RGSwear in daily practice? Why / why not
- Which one did you like the most/the least? and why
- What way would be most suitable for you to get motivated to do exercises?

**AISN Technology (~ 30 min)**

GENERIC QUESTIONS

**Awareness and Perception of** AI

- Are you familiar with the concept of Artificial Intelligence (AI)? (Yes/No
- How would you define Artificial Intelligence in your own words
- Do you believe Artificial Intelligence can play a role in clinical rehabilitation
- If yes, how do you think AI can be beneficial in clinical rehabilitation
- If no, what concerns or reservations do you have about the use of AI in clinical rehabilitation

**Experience with AI in** Rehabilitation

- Have you ever used or interacted with AI-based tools or technologies in a healthcare or rehabilitation setting? If yes, please describe your experience
- If not would you be willing to use AI-based tools or technologies in your rehabilitation process?

SPECIFIC QUESTIONS

**AI integrated into the RGS**

- How do you feel interacting with rehabilitation technology that uses Artificial Intelligence (AI) to adjust the treatment in order to be patient-specific
- How do you feel about clinicians using AI-based recommendations for patients' rehabilitation

**Expectations and** Preferences

- What specific features or AI-based capabilities would you like to see in clinical rehabilitation with RGS
- How important is it for you to have human oversight or involvement when using AI-based tools in your rehabilitation

**Concerns and** Limitations

- What concerns, if any, do you have about the use of AI in clinical rehabilitation
- Are there any ethical considerations that should be taken into account when implementing AI in rehabilitation programmes

**Impact and** Effectiveness

- In your opinion, how do you think AI-based tools could improve the effectiveness of clinical rehabilitation programs such as RGS
- What potential challenges do you foresee in integrating AI into clinical rehabilitation practices

**Future** Adoption

- Would you recommend AI-based tools to others undergoing clinical rehabilitation?
- What factors would influence your decision to recommend or not recommend AI-based tools to others

**Additional Comments**

- Is there anything else you would like to share regarding the use of AI in clinical rehabilitation
- What are the advantages, risks and impact of this type of tool in your life
- In your opinion, what is needed for a patient to use AISN in rehabilitation post-stroke? And the clinicians

**AI-based decision-support module for Clinicians (only for clinicians) (~ 30 min)**

**MIMS (Medical Information Management System)**

This platform allows healthcare professionals to remotely manage, prescribe activities and daily training, and follow up on patients' performance and adherence via the MIMS dashoboard and objective reports, such as, exercises prescriptions, patients' adherence, AI-based diagnostics, and prognostics predictions

- Do you have previous experience with this kind of tool
- How was your experience using MIMS? Did you find them easy to use? Or did you have any problems
- In your opinion, what kind of indicators or metrics would be useful for remote monitoring of patients?
- How often do you follow up remotely with your patients about their adherence to the exercise's prescriptions?
- What other kind of recommendations do you want to receive from AISN
- Do you find it useful for your daily practice? Why / Why not
- What do you consider the biggest strength of MIMS for prescription and its reports
- What areas do you think could be improved

**S2. Description of RGSapp exercises**

| **MEMOSEQ**   | In this exercise, the user is presented with a series of everyday objects to be selected according to specific sequences and parameters, such as their size and shape, and order of appearance. |
| --- | --- |
| **BLOBS**   | In this exercise, the user has to perform *finger pinch movements* to merge or split colour-coded shapes while making simple math operations**.** |
| **SHELVES (AR)**   | In this exercise, the user is presented with a series of objects that need to be memorized in sequence. These objects can be reached and removed from a shelf according to the proposed order. |
| **DRIVING**   | **Motor components**   - Rotation movements (holding the phone with 2 hands) - Reaching movement   Grasping (full arm) |

| **Spheroids**  | In this exercise, a series of colored spheres approach the user from the horizon, and the user must stop them with their hands to score points. The left and right balls must be touched with the left and right arms, respectively. |
| --- | --- |
| **Clean the Table**  | A table is covered with white cubes that the patient must throw off the edges of the table. The round ends when all the cubes have been removed and a new table is presented. The tables vary in size and shape. |
| **Pinball**  | Balls of different colors roll along an obstacle course. At the top center of the screen, the user sees a sphere in the target color. Only the spheres in the target color must be intercepted; the other spheres must be avoided. |
| **Hockey**  | The user must hit the hockey pucks on the rink before they disappear to score goals. Once hit, if the puck does not go into the goal, it will bounce and can be hit again. To make new pucks appear, the user must return their hands to the starting position (with elbows bent), as indicated by a pair of hands displayed at the bottom of the screen. |

**S4. Reports in detail all the main demographic characteristics of all patients who participated in the FG**

| **Country** | **Gender** | **Age** | **Time since stroke (months)** | **Occupation** | **Education** | **Tech knowledge** | **Digital competency** |
| --- | --- | --- | --- | --- | --- | --- | --- |
| Italy | Male | 79 | 48 | retired | School | good/mobile | basic |
| Italy | Female | 71 | 12 | full-time | Higher | good/mobile | medium |
| Italy | Male | 51 | 7 | full-time | Professional | good/mobile | medium |
| Italy | Male | 79 | 24 | retired | School | very low | very low |
| Italy | Female | 81 | 28 | retired | Higher | very low | medium |
| Italy | Male | 57 | 9 | retired | School | good/mobile | basic |
| Italy | Male | 77 | 24 | retired | Professional | good/mobile | basic |
| Italy | Male | 70 | 360 | retired | Professional | very low | basic |
| Italy | Male | 81 | 72 | retired | School | good/mobile | medium |
| Spain | Female | 86 | 48 | Other | Professional | Mobile Apps | Basic |
| Spain | Female | 70 | 120 | Retired | School | Mobile Apps | Basic |
| Spain | Female | 80 | 18 | Retired | School | Mobile Apps | Basic |
| Spain | Male | 86 | 24 | Retired | Higher | Mobile Apps | Medium |
| Spain | Female | 80 | 72 | Retired | Professional | Mobile Apps/ RGS | Medium |
| France | Male | 61 | 1,5 | Retired | Professional | VR, smartwatch | Proficient |
| France | Female | 78 | 1,5 | Retired | Professional | mobile app | Proficient |
| France | Male | 33 | 30 |  |  | mobile app, smartwatch | Medium |
| France | Male | 62 | 15,5 | Retired |  | none | Basic |
| France | Female | 56 | 1 | teacher | Professional | VR, smartwatch | Medium |
| France | Female | 62 | 192 | Retired | Professional | mobile app | Medium |
| France | Female | 71 | 36 |  | Professional | mobile app | Proficient |
| France | Male | 70 | 48 |  | School | none |  |
| Romania | Male | 48 | 2 | full-time | Professional | Good/mobile | Basic |
| Romania | Male | 52 | Unknown | full-time | Higher | Good/mobile | Medium |
| Romania | Male | 45 | 4 | full-time | School | Mobile Apps | Basic |
| Romania | Male | 63 | 20 | full-time | Higher | Mobile Apps | Basic |
| Romania | Male | 61 | 6 | full-time | School | Very low | Very low |

**S5. Reports in detail all the main demographic characteristics of all caregivers who participated in the FG**

| **Country** | **Gender** | **Age** | **Occupation** | **Education** | **Tech**  **knowledge** | **Digital competency** |
| --- | --- | --- | --- | --- | --- | --- |
| Italy | Female | 59 | full-time | Professional | very low | basic |
| Italy | Female | 69 | Retired | Higher | good/mobile | proficient |
| Italy | Female | 29 | full-time | Higher | good/mobile, pc | proficient |
| Italy | Female | 71 | Retired | School | none | basic |
| Italy | Male | 20 | student | Higher | very good/mobile, pc | proficient |
| Italy | Female | 75 | Retired | School | none | basic |
| Italy | Female | 55 | full-time | Professional | good/mobile, pc | proficient |
| Italy | Female | 79 | Retired | Higher | low/mobile | basic |
| Spain | Male | 44 | Full/Part-time employee | Higher | Mobile apps | Basic |
| Spain | Female | 59 | Full/Part-time employee | Professional | Mobile apps | Medium |
| Spain | Female | 81 | Retired | School | Mobile apps | Medium |
| France | Female | 64 | Nurse retired | Professional | Mobile apps, smartwatch | Medium |
| France | Male | 74 | Retired | Professional | None | Basic |
| France | Male | 75 | Retired | Higher | Virtual Reality, smartwatch | Medium |
| France | Male | 62 | Teacher | Higher | smartwatch | Medium |
| France | Female | 71 | Retired | Professional | None | Medium |
| France | Male | 64 | Retired | Professional | augmented reality | Medium |

**S6. Reports in details all the main demographic characteristics of all healthcare professionals who participated in the FG**

| **Country** | **Age** | **Gender** | **Occupation** | **Education** | **Role** | **Years**  **ex** | **Tech**  **knowledge** | **Digital**  **competency** |
| --- | --- | --- | --- | --- | --- | --- | --- | --- |
| Italy | 28 | Female | full time | Higher | speech therapist | 2 | basic knowledge on VR/apps | medium |
| Italy | 25 | Female | full time | Higher | speech therapist | 1 | basic knowledge on VR/apps | basic |
| Italy | 35 | Female | full time | Higher | geriatrician | 10 | VR/apps | basic |
| Italy | 31 | Female | other | Higher | physician | 3 | other | medium |
| Italy | 34 | Male | full time | Higher | physiotherapist | 2 | VR/apps | medium |
| Italy | 27 | Female | full time | Higher | speech therapist | 4 | apps | medium |
| Italy | 24 | Male | full time | Higher | speech therapist | 2 | VR/apps | medium |
| Italy | 26 | Female | full time | Higher | research assistant | 2 | apps/other (telerehabilitation) | medium |
| Italy | 29 | Female | full time | Higher | psychologist | 3 | apps | medium |
| Spain | 24 | Male | Full/Part-time employee | Higher | Physiotherapist | 2 | Virtual reality/Mobile Apps/ RGS/Other Recommendation systems | Proficient |
| Spain | 32 | Female | Full/Part-time employee | Higher | Physiotherapist | 9 | Virtual reality/Mobile Apps/ Other Recommendation systems | Proficient |
| Spain | 40 | Female | Full/Part-time employee | Higher | Internal medicine | 9 | Virtual reality/Mobile Apps | Proficient |
| Spain | 56 | Male | Full/Part-time employee | Higher | Neurologist | 27 | Virtual reality/Mobile Apps  RGS/Others Recommendation systems | Proficient |
| Spain | 46 | Male | Full/Part-time employee | Higher | Internal medicine | 16 | Virtual reality/Mobile Apps  RGS | Proficient |
| France | 22 | Female | full time | Higher | occupational therapist | 1 | VR, AR, mobile app, smartwatch, e-health | medium |
| France | 24 | Female | full time | Higher | occupational therapist | 1 | VR, AR, mobile app, smartwatch | medium |
| France | 22 | Male | full time | Higher | occupational therapist | 1 | VR, smartwatch | basic |
| France | 21 | Female | full time | Higher | occupational therapist | 1 | VR, smartwatch, IVS 3 ChatGPT | medium |
| France | 22 | Female | full time | Higher | occupational therapist | 1 | VR, mobile app, smartwatch | basic |
| France | 29 | Male | full time | Higher | Physiotherapist | 7 | VR, AR, mobile app, smartwatch | Proficient |
| France | 39 | Male | full time | Higher | Physiotherapist | 17 | VR, AR, mobile app, smartwatch | medium |
| France | 31 | Male | full time | Higher | Physiotherapist | 6 | VR, AR, mobile app, smartwatch | Proficient |
| France | 48 | Male | full time | Higher | Physiotherapist | 20 | VR, AR, mobile app, smartwatch | medium |
| France | 25 | Male | full time | Higher | Physiotherapist | 3 | VR, mobile app, smartwatch | Proficient |
| France | 27 | Female | full time | Higher | Neuropsychologist | 4,5 | VR, mobile app, smartwatch | medium |
| France | 45 | Male | full time | Higher | Physiotherapist | 4 | mobile app, smartwatch | medium |
| France | 46 | Female | full time | Higher | occupational therapist | 23 | mobile app, smartwatch, robotic rehabilitation devices | Proficient |

**S7. Summary of Codes from Patients (RGSapp and RGSclinic)**

| **Nº** | **RGSapp** **code** | **n = 8 France** | **n = 9 Italy** | **n = 5 Spain** | **n = 5 Romania** | **n = 27 Total** |
| --- | --- | --- | --- | --- | --- | --- |
| 1 | usefulness and complementarity | 8 | 9 | 5 | 5 | **27** |
| 2 | no good detection | 4 | 3 | 3 | 4 | **14** |
| 3 | a minimum of digital literacy needed | 3 | 9 | 5 | 5 | **22** |
| 4 | need of family support | 0 | 9 | 3 | 4 | **16** |
| 5 | need of professional support | 3 | 9 | 5 | 3 | **20** |
| 6 | human interaction needed | 8 | 9 | 5 | 4 | **26** |
| 7 | add better instructions | 2 | 7 | 3 | 5 | **17** |
| 8 | personal preferences and adaptation | 6 | 9 | 5 | 3 | **23** |
| 9 | regularity rather than quantity of exercises | 4 | 3 | 0 | 2 | **9** |
| 10 | fatigue and personal temperament | 5 | 4 | 0 | 2 | **11** |
| 11 | positive feedback about monitoring | 4 | 9 | 5 | 4 | **22** |
| 12 | safety feeling | 4 | 9 | 0 | 3 | **16** |
| 13 | lack of digital technology | 4 | 7 | 3 | 5 | **19** |
| 14 | need of a initial training to use | 4 | 9 | 3 | 5 | **21** |
| 15 | connection and support issues | 5 | 3 | 5 | 0 | **13** |
| 16 | fear of isolation | 8 | 4 | 5 | 2 | **19** |
| 17 | adaptation time nedeed | 4 | 9 | 2 | 3 | **18** |
| 18 | security risks to personal data | 0 | 8 | 2 | 1 | **11** |

| **Nº** | **RGSclinic code** | **n = 8 France** | **n = 9 Italy** | **n = 5 Spain** | **n = 5 Romania** | **n = 27 Total** |
| --- | --- | --- | --- | --- | --- | --- |
| 1 | positive feedback | 0 | 9 | 4 | 4 | **17** |
| 2 | need for a big screen | 2 | 6 | 3 | 5 | **16** |
| 3 | usefulness and complementarity | 8 | 9 | 4 | 4 | **25** |

**S8. Summary of Codes from Caregivers (RGSapp and RGSclinic)**

| **Nº** | **RGSapp code** | **n = 6 France** | **n = 8 Italy** | **n = 3 Spain** | **n = 17 Total** |
| --- | --- | --- | --- | --- | --- |
| 1 | positive feedback | 6 | 5 | 3 | **14** |
| 2 | unclear instructions | 0 | 8 | 1 | **9** |
| 3 | poor self-perception of their technological skills | 0 | 0 | 1 | **1** |
| 4 | not very intuitive | 2 | 2 | 1 | **5** |
| 5 | Importance of motivation and personal progress | 2 | 8 | 3 | **13** |
| 6 | concerns about application crashes | 5 | 3 | 3 | **11** |
| 7 | personalization of the treatment adapted to the patients’ needs | 0 | 4 | 3 | **7** |
| 8 | human interaction needed | 3 | 8 | 3 | **14** |
| 9 | concern about budget and autonomy | 6 | 8 | 1 | **15** |
| 10 | technical support needed | 6 | 5 | 3 | **14** |
| 11 | visual instructions | 0 | 2 | 3 | **5** |
| 12 | not infantilising games | 0 | 2 | 3 | **5** |

| **Nº** | **RGSclinic code** | **n = 6 France** | **n = 8 Italy** | **n = 3 Spain** | **n = 17 Total** |
| --- | --- | --- | --- | --- | --- |
| 1 | positive feedback | 5 | 8 | 3 | **16** |
| 2 | no good detection | 1 | 2 | 3 | **6** |
| 3 | proposing a game incorporating a keyword | 3 | 0 | 0 | **3** |

**S9. Summary of Codes from Healthcare Professionals (RGSapp and RGSclinic)**

| **Nº** | **RGSapp** **code** | **n = 13 France** | **n = 9 Italy** | **n = 5 Spain** | **n = 27 Total** |
| --- | --- | --- | --- | --- | --- |
| 1 | simple and intuitive use | 13 | 9 | 4 | **26** |
| 2 | positive initial impression | 0 | 9 | 5 | **14** |
| 3 | general need for improvement | 6 | 8 | 5 | **19** |
| 4 | concerns about readability | 5 | 6 | 0 | **11** |
| 5 | as a complement care | 13 | 8 | 1 | **22** |
| 6 | need for support | 10 | 9 | 3 | **22** |
| 7 | risks of tech addiction | 3 | 7 | 0 | **7** |
| 8 | need of specific training | 7 | 9 | 5 | **21** |
| 9 | importance of clear instructions | 4 | 6 | 5 | **15** |
| 10 | professional and family support needed | 2 | 9 | 5 | **16** |
| 11 | link between protocols and daily activities | 10 | 4 | 5 | **19** |
| 12 | there should be human - patient contact | 13 | 9 | 5 | **27** |
| 13 | concern about bad exercise performance | 0 | 7 | 5 | **12** |

| **Nº** | **RGSclinic code** | **n = 13 France** | **n = 9 Italy** | **n = 5 Spain** | **n = 27 Total** |
| --- | --- | --- | --- | --- | --- |
| 1 | need for support | 8 | 9 | 5 | **22** |
| 2 | technological barrier | 8 | 9 | 4 | **21** |
| 3 | adaptation to the patient's preferences | 2 | 7 | 5 | **14** |
| 4 | concerns about maintaining adherence to treatment | 0 | 7 | 5 | **12** |
| 5 | very positive feedback on home installation | 0 | 4 | 4 | **8** |
| 6 | concern about bad exercise performance | 7 | 9 | 5 | **21** |
| 7 | balance of home and clinic-based rehabilitation | 0 | 7 | 5 | **12** |
| 8 | possibility of incorporate VR | 0 | 0 | 1 | **1** |

**S10. Summary of Codes from Healthcare Professionals on the MIMS Platform**

| **Nº** | **MIMS code** | **n = 13 France** | **n = 9 Italy** | **n = 5 Spain** | **n = 27 Total** |
| --- | --- | --- | --- | --- | --- |
| 1 | need of an initial training to use | 8 | 2 | 5 | **15** |
| 2 | technical problems | 6 | 0 | 5 | **11** |
| 3 | intuitive and simple interface | 5 | 7 | 5 | **17** |
| 4 | make a user-friendly interface | 2 | 3 | 4 | **9** |
| 5 | positive initial impression | 3 | 9 | 5 | **17** |
| 6 | positive feedback on patient monitoring | 0 | 9 | 5 | **14** |
| 7 | extend functionalities needed | 8 | 4 | 3 | **15** |

**S11. Mean SUS item scores by stakeholder group**

| **SUS item** | **Stroke survivors (n = 27)** | **Caregivers (n = 17)** | **Healthcare professionals (n = 27)** | **All (n = 71)** |
| --- | --- | --- | --- | --- |
| 1. I think that I would like to use this system frequently (+) | 3.41 | 4.47 | 3.81 | 3.82 |
| 2. I found the system unnecessarily complex (−) | 2.93 | 1.65 | 1.89 | 2.23 |
| 3. I thought the system was easy to use (+) | 3.63 | 4.06 | 3.78 | 3.79 |
| 4. I think that I would need the support of a technical person to be able to use this system (−) | 3.37 | 2.59 | 2.30 | 2.77 |
| 5. I found the various functions in this system were well integrated (+) | 3.96 | 4.29 | 4.11 | 4.10 |
| 6. I thought there was too much inconsistency in this system (−) | 2.63 | 1.76 | 1.44 | 1.97 |
| 7. I would imagine that most people would learn to use this system very quickly (+) | 3.59 | 3.76 | 3.15 | 3.46 |
| 8. I found the system very cumbersome to use (−) | 2.85 | 1.76 | 1.96 | 2.25 |
| 9. I felt very confident using the system (+) | 3.56 | 4.24 | 3.67 | 3.76 |
| 10. I needed to learn a lot of things before I could get going with this system (−) | 2.78 | 2.82 | 1.85 | 2.44 |

*Items are rated from 1 (strongly disagree) to 5 (strongly agree). (+) positively worded items: higher is better; (−) negatively worded items: lower is better. Stroke survivors and caregivers rated the patient-facing RGS; healthcare professionals rated the MIMS/RGS system. Two unanswered items (one stroke survivor, item 5; one caregiver, item 3) were excluded from the item means.*

**S12. Individual SUS responses and total scores of all participants**

| **Participant** | **Country** | **Q1** | **Q2** | **Q3** | **Q4** | **Q5** | **Q6** | **Q7** | **Q8** | **Q9** | **Q10** | **SUS total** |
| --- | --- | --- | --- | --- | --- | --- | --- | --- | --- | --- | --- | --- |
| SS-IT-01 | Italy | 5 | 5 | 3 | 5 | 5 | 5 | 3 | 4 | 4 | 4 | 42.5 |
| SS-IT-02 | Italy | 1 | 4 | 1 | 5 | 5 | 2 | 5 | 4 | 1 | 5 | 32.5 |
| SS-IT-03 | Italy | 4 | 3 | 4 | 2 | 3 | 2 | 4 | 2 | 2 | 3 | 62.5 |
| SS-IT-04 | Italy | 5 | 1 | 5 | 1 | 5 | 1 | 5 | 1 | 5 | 1 | 100.0 |
| SS-IT-05 | Italy | 4 | 2 | 5 | 1 | 4 | 5 | 5 | 2 | 3 | 3 | 70.0 |
| SS-IT-06 | Italy | 3 | 3 | 3 | 3 | 3 | 3 | 3 | 3 | 3 | 3 | 50.0 |
| SS-IT-07 | Italy | 3 | 2 | 2 | 4 | 3 | 2 | 2 | 3 | 3 | 3 | 47.5 |
| SS-IT-08 | Italy | 3 | 2 | 4 | 5 | 4 | 2 | 2 | 3 | 3 | 4 | 50.0 |
| SS-IT-09 | Italy | 2 | 4 | 3 | 5 | 5 | 1 | 3 | 2 | 4 | 5 | 50.0 |
| SS-ES-01 | Spain | 5 | 2 | 2 | 4 | 4 | 2 | 4 | 2 | 3 | 4 | 60.0 |
| SS-ES-02 | Spain | 5 | 1 | 4 | 4 | 4 | 2 | 4 | 4 | 3 | 3 | 65.0 |
| SS-ES-03 | Spain | 1 | 4 | 2 | 2 | – | 5 | 3 | 2 | 2 | 1 | 41.7* |
| SS-ES-04 | Spain | 5 | 3 | 5 | 5 | 5 | 1 | 5 | 5 | 4 | 3 | 67.5 |
| SS-ES-05 | Spain | 5 | 1 | 5 | 5 | 5 | 1 | 5 | 1 | 5 | 3 | 85.0 |
| SS-FR-01 | France | 3 | 1 | 5 | 3 | 3 | 1 | 3 | 1 | 5 | 2 | 77.5 |
| SS-FR-02 | France | 4 | 3 | 3 | 5 | 4 | 4 | 4 | 4 | 5 | 5 | 47.5 |
| SS-FR-03 | France | 1 | 5 | 5 | 2 | 5 | 5 | 2 | 5 | 5 | 1 | 50.0 |
| SS-FR-04 | France | 4 | 3 | 5 | 5 | 5 | 1 | 5 | 1 | 5 | 1 | 82.5 |
| SS-FR-05 | France | 4 | 2 | 4 | 4 | 4 | 2 | 3 | 3 | 4 | 1 | 67.5 |
| SS-FR-06 | France | 4 | 1 | 4 | 4 | 5 | 1 | 3 | 1 | 5 | 1 | 82.5 |
| SS-FR-07 | France | 3 | 5 | 3 | 2 | 2 | 1 | 3 | 2 | 3 | 3 | 52.5 |
| SS-FR-08 | France | 1 | 4 | 3 | 1 | 4 | 2 | 3 | 5 | 1 | 1 | 47.5 |
| SS-RO-01 | Romania | 4 | 5 | 5 | 5 | 1 | 5 | 5 | 5 | 5 | 5 | 37.5 |
| SS-RO-02 | Romania | 4 | 4 | 2 | 1 | 5 | 4 | 5 | 1 | 4 | 1 | 72.5 |
| SS-RO-03 | Romania | 5 | 5 | 4 | 2 | 5 | 5 | 4 | 5 | 5 | 5 | 52.5 |
| SS-RO-04 | Romania | 3 | 2 | 5 | 5 | 3 | 4 | 3 | 4 | 3 | 3 | 47.5 |
| SS-RO-05 | Romania | 1 | 2 | 2 | 1 | 2 | 2 | 1 | 2 | 1 | 1 | 47.5 |
| CG-IT-01 | Italy | 5 | 2 | 5 | 4 | 5 | 2 | 5 | 2 | 4 | 4 | 75.0 |
| CG-IT-02 | Italy | 4 | 1 | 4 | 3 | 5 | 1 | 3 | 2 | 5 | 4 | 75.0 |
| CG-IT-03 | Italy | 5 | 2 | 5 | 1 | 5 | 2 | 5 | 1 | 5 | 1 | 95.0 |
| CG-IT-04 | Italy | 5 | 2 | 5 | 4 | 5 | 3 | 4 | 3 | 3 | 5 | 62.5 |
| CG-IT-05 | Italy | 3 | 1 | 4 | 1 | 4 | 1 | 5 | 1 | 3 | 1 | 85.0 |
| CG-IT-06 | Italy | 5 | 4 | 2 | 5 | 4 | 5 | 4 | 3 | 4 | 5 | 42.5 |
| CG-IT-07 | Italy | 5 | 1 | 4 | 1 | 4 | 1 | 4 | 2 | 4 | 1 | 87.5 |
| CG-IT-08 | Italy | 4 | 1 | 3 | 1 | 3 | 1 | 4 | 1 | 5 | 5 | 75.0 |
| CG-ES-01 | Spain | 5 | 1 | 5 | 2 | 5 | 1 | 4 | 1 | 5 | 2 | 92.5 |
| CG-ES-02 | Spain | 5 | 1 | 5 | 1 | 5 | 2 | 2 | 3 | 3 | 5 | 70.0 |
| CG-ES-03 | Spain | 5 | 3 | 2 | 4 | 5 | 1 | 3 | 1 | 3 | 4 | 62.5 |
| CG-FR-01 | France | 4 | 1 | 4 | 3 | 4 | 1 | 3 | 2 | 5 | 1 | 80.0 |
| CG-FR-02 | France | 5 | 1 | 5 | 1 | 4 | 1 | 2 | 1 | 5 | 1 | 90.0 |
| CG-FR-03 | France | 4 | 2 | – | 3 | 4 | 1 | 4 | 2 | 5 | 4 | 72.2* |
| CG-FR-04 | France | 5 | 1 | 3 | 5 | 3 | 1 | 4 | 1 | 4 | 1 | 75.0 |
| CG-FR-05 | France | 3 | 3 | 4 | 3 | 4 | 4 | 5 | 2 | 4 | 3 | 62.5 |
| CG-FR-06 | France | 4 | 1 | 5 | 2 | 4 | 2 | 3 | 2 | 5 | 1 | 82.5 |
| HP-IT-01 | Italy | 3 | 2 | 5 | 2 | 4 | 2 | 3 | 2 | 4 | 2 | 72.5 |
| HP-IT-02 | Italy | 4 | 1 | 3 | 4 | 4 | 1 | 3 | 2 | 4 | 1 | 72.5 |
| HP-IT-03 | Italy | 5 | 2 | 3 | 2 | 4 | 1 | 5 | 2 | 3 | 1 | 80.0 |
| HP-IT-04 | Italy | 3 | 3 | 3 | 1 | 3 | 2 | 2 | 4 | 2 | 2 | 52.5 |
| HP-IT-05 | Italy | 3 | 2 | 3 | 4 | 3 | 1 | 3 | 2 | 3 | 4 | 55.0 |
| HP-IT-06 | Italy | 3 | 4 | 2 | 3 | 3 | 2 | 3 | 4 | 4 | 3 | 47.5 |
| HP-IT-07 | Italy | 2 | 3 | 3 | 3 | 4 | 2 | 2 | 3 | 3 | 2 | 52.5 |
| HP-IT-08 | Italy | 5 | 2 | 3 | 2 | 4 | 2 | 3 | 3 | 4 | 2 | 70.0 |
| HP-IT-09 | Italy | 4 | 2 | 3 | 2 | 4 | 1 | 3 | 1 | 4 | 2 | 75.0 |
| HP-ES-01 | Spain | 4 | 2 | 5 | 2 | 5 | 1 | 5 | 1 | 4 | 3 | 85.0 |
| HP-ES-02 | Spain | 5 | 1 | 4 | 1 | 4 | 1 | 3 | 1 | 4 | 1 | 87.5 |
| HP-ES-03 | Spain | 5 | 2 | 4 | 1 | 5 | 1 | 3 | 1 | 4 | 2 | 85.0 |
| HP-ES-04 | Spain | 5 | 1 | 5 | 4 | 5 | 1 | 4 | 1 | 4 | 3 | 82.5 |
| HP-ES-05 | Spain | 4 | 2 | 3 | 3 | 4 | 1 | 4 | 2 | 4 | 2 | 72.5 |
| HP-FR-01 | France | 4 | 1 | 5 | 2 | 5 | 1 | 3 | 2 | 4 | 2 | 82.5 |
| HP-FR-02 | France | 3 | 2 | 5 | 4 | 4 | 2 | 2 | 2 | 4 | 2 | 65.0 |
| HP-FR-03 | France | 4 | 1 | 4 | 1 | 5 | 1 | 3 | 2 | 5 | 1 | 87.5 |
| HP-FR-04 | France | 4 | 1 | 4 | 3 | 3 | 2 | 3 | 2 | 3 | 2 | 67.5 |
| HP-FR-05 | France | 3 | 2 | 4 | 4 | 4 | 1 | 3 | 1 | 3 | 2 | 67.5 |
| HP-FR-06 | France | 4 | 2 | 4 | 1 | 5 | 1 | 4 | 1 | 4 | 2 | 85.0 |
| HP-FR-07 | France | 5 | 2 | 5 | 1 | 5 | 3 | 5 | 1 | 4 | 1 | 90.0 |
| HP-FR-08 | France | 4 | 2 | 4 | 2 | 5 | 1 | 2 | 2 | 5 | 1 | 80.0 |
| HP-FR-09 | France | 4 | 1 | 4 | 3 | 5 | 1 | 4 | 1 | 4 | 1 | 85.0 |
| HP-FR-10 | France | 4 | 2 | 4 | 1 | 4 | 1 | 3 | 1 | 2 | 1 | 77.5 |
| HP-FR-11 | France | 2 | 2 | 3 | 2 | 3 | 2 | 2 | 2 | 3 | 1 | 60.0 |
| HP-FR-12 | France | 3 | 3 | 3 | 3 | 3 | 3 | 3 | 3 | 3 | 3 | 50.0 |
| HP-FR-13 | France | 4 | 1 | 4 | 1 | 4 | 1 | 2 | 4 | 4 | 1 | 75.0 |

*Answers range from 1 (strongly disagree) to 5 (strongly agree). Participant codes were created for this table: SS = stroke survivor, CG = caregiver, HP = healthcare professional; IT = Italy, ES = Spain, FR = France, RO = Romania. SUS total scores were computed following Brooke (1996). – = unanswered item; * total score prorated from the nine answered items.*
